# Fitness consequences of sex chromosome aneuploidy in *Drosophila melanogaster*

**DOI:** 10.1371/journal.pgen.1011703

**Published:** 2025-06-03

**Authors:** Elizabeth R. Makovec, Caitlin C. Kestell, Kayla K. Janke, Ethan J. Carter, Aaron P. Ragsdale, Nathaniel P. Sharp

**Affiliations:** 1 Department of Genetics, University of Wisconsin-Madison, Madison, Wisconsin, United States of America; 2 Department of Integrative Biology, University of Wisconsin-Madison, Madison, Wisconsin, United States of America.; North Dakota State University, UNITED STATES OF AMERICA

## Abstract

Of the array of spontaneous mutations that can occur, changes to chromosome number may have the greatest impact on the evolutionary potential of populations and the condition of affected individuals. Chromosomal nondisjunction resulting in aneuploidy is found across eukaryotes, but the consequences of such karyotypic variation have not been widely explored. In the fruit fly *Drosophila melanogaster*, aneuploid females with an XXY karyotype can arise through nondisjunction, inheriting a Y chromosome from their male parent. While the Y chromosome contains few genes, the large amount of heterochromatic DNA it contains can substantially alter genome-wide gene expression in females. We conducted a series of experiments to understand how sex chromosome aneuploidy alters key traits in affected females and their progeny. In the same genetic background, we also determined the rate at which this karyotype appears spontaneously and its standing frequency. We found that XXY females largely resembled XX females, but experienced size and fecundity benefits when receiving a male-transmitted Y chromosome. However, XYY males produced by aneuploid females experienced reduced viability, limiting the standing frequency of aneuploidy at mutation-selection equilibrium. Our findings demonstrate that aneuploid flies are not too rare in laboratory populations, but that the effects of this karyotypic diversity depend on sex and parent of origin.

## Author summary

An important error that can occur during reproduction is the gain or loss of an entire chromosome, and such genomic changes are often detrimental for affected progeny. We investigated the origination and consequences of abnormal Y chromosome copy number in fruit flies. In this organism, females are typically XX, but can also inherit a Y chromosome in rare cases. Such XXY females are known to have altered gene expression, but little is known about their reproductive traits and the persistence of extra Y chromosomes. We find that XXY females are comparable to XX females in some respects, but show enhanced productivity when they inherit a Y chromosome from a male. However, these females will also produce XYY male offspring, which we find have reduced viability, limiting the spread of extra Y chromosomes through the population. Combining evolutionary models with our measures of XXY origination rate, we conclude that the reduced viability of XYY males is sufficient to explain the observed standing frequency of XXY in a laboratory population. Our study provides insight into the evolutionary and genetic processes governing an important aspect of genomic variation.

## Introduction

Genetic variation introduced by spontaneous mutation is the raw material for evolution. While most mutations involve few nucleotides, much larger genomic changes also occur, including the gain or loss of entire chromosomes. During meiosis, improper chromosome segregation (nondisjunction) can result in progeny with atypical karyotypes (aneuploidy). Such variation is likely to be deleterious in many cases but could also be a source of evolutionary novelty [1–5]. In humans, aneuploidy is a major cause of miscarriage and disease [6–8]. In contrast, yeast appear to be relatively tolerant of aneuploidy [9,10], with karyotype variation found among natural isolates [11] and implicated in many laboratory studies of adaptation [12]. In the fruit fly *Drosophila melanogaster*, aneuploidy for a major autosome results in embryonic lethality, whereas aneuploidy for the small fourth chromosome or the Y chromosome does not [13,14]. Indeed, sex chromosome aneuploidy in flies has been used for more than a century to establish and study key principles of genetics, including the chromosome theory of heredity [15,16]. However, the impact and frequency dynamics of such karyotypic variation is not yet clear. Here, we describe experiments with *D. melanogaster* designed to explore this phenomenon quantitatively, to better understand the evolutionary consequences of nondisjunction. We consider the rate at which sex chromosome aneuploidy appears spontaneously in a laboratory population, how aneuploidy is transmitted between generations, the fitness consequences of that variation, and the standing frequency of aneuploids.

In diploid *D. melanogaster*, sex is determined by the number of X chromosomes, where females have two and males have one [17]. Sex chromosome nondisjunction is believed to occur in approximately one out of every thousand meioses, and results in some progeny that are inviable or sterile [14]. Specifically, nondisjunction in females results in XX and ∅ (nullo-X) gametes, which give rise to XXX, XXY, X∅ and Y∅ zygotes when combined with normal male gametes (S1 Fig); nondisjunction in males results in XY and ∅ gametes, which give rise to XXY and X∅ zygotes when combined with normal female gametes (S1 Fig). XXX females have low viability and fertility, Y∅ is embryonic lethal, and X∅ males are viable but sterile; however, XXY flies are viable, phenotypically “normal” females [14,15]. Importantly, “supernumerary” Y chromosomes can be transmitted to the next generation, as XXY females will produce XXY female and XYY male progeny (S2 Fig); our study is centered on these karyotypes.

The Y chromosome in this species is entirely heterochromatic and contains few genes, including male fertility factors and an array of ribosomal DNA (rDNA) repeats [14]. This chromosome is also relatively large, making up about 11% of the diploid nuclear genome in XY males and about 10% in XXY females [18], and comprised mainly of repetitive satellite sequences [19]. A supernumerary Y chromosome therefore represents a significant deviation from typical genome content but little change in terms of protein coding gene content. There is evidence that Y chromosome copy number can affect mating behavior [20–24], aging [25], and genome-wide chromatin states [26–29]. In XXY females, Y-linked genes are not transcribed, but the presence of Y-linked heterochromatin alters gene expression patterns throughout the genome, including genes implicated in sexual behavior and sex-specific tissues [30]. Given these findings, we hypothesized that XXY females would differ from XX females in fitness-related traits. More broadly, we considered how “mutation-selection balance” dynamics might apply in this circumstance, where mutation (nondisjunction) introduces the karyotypic variant and purifying selection limits its frequency, resulting in a non-zero expected frequency of the mutant karyotype at equilibrium.

To understand the dynamics of sex chromosome aneuploidy, we sought to address the following questions: (i) At what rate do XXY flies arise spontaneously, and how do females versus males contribute to nondisjunction events? (ii) How do supernumerary Y chromosomes affect fitness in males and females? (iii) What is the standing frequency of the XXY karyotype, and is it consistent with the observed levels of mutation and selection?

We found a sex bias in sex chromosome nondisjunction, with the XXY karyotype arising primarily through female nondisjunction. Surprisingly, the resulting XXY females had fitness similar to that of XX females, or even greater in some respects, but XYY males exhibited reduced viability. Using population genetic models, we found that viability selection against XYY, along with the inviability of more complex karyotypes, was sufficient to explain the low standing frequency of XXY females we observed. We conclude that the XXY karyotype is not particularly harmful to flies, but its spread is prevented by selection against related karyotypes. Although we studied a lab population, our findings are relevant for understanding the likely paths of genome evolution in nature, particularly given evidence that the XXY karyotype can become common in certain wild populations [31].

## Results

### Key methods summary

In order to identify and study females with the XXY karyotype, we used fly strains with Y chromosomes carrying visible marker alleles, as in previous studies [21,25,27,30,32–34]; we therefore also considered any potential effect of the markers themselves. An exception is our assay of XXY standing frequency, which involved an un-marked laboratory population, where we instead apply a molecular assay and test crosses to identify karyotypes. We assessed the effects of “free” Y chromosomes (i.e., not attached to an X chromosome), but utilized attached X-Y stocks for certain crosses. The cross designs used in each assay are illustrated in S1-S11 Figs, with corresponding datasets given in S1-S16 Tables.

### Spontaneous nondisjunction

We first considered the rate of spontaneous nondisjunction leading to XXY females. Previous estimates of this rate exist (reviewed in [14]), but given evidence for genetic variation in nondisjunction [35,36], we obtained estimates for our lab population to ensure an appropriate comparison with our other measurements, particularly the standing frequency of the XXY karyotype. Our estimates of non-disjunction rates are based on two different assays (S1 Fig and S1 Table; S3 Fig and S2 Table), involving 38178 offspring in total, where “exceptional” (aneuploid) progeny can be phenotypically identified; one of our experiments also allowed the parent of origin of nondisjuction events to be determined. We did not find a significant difference between assays in the frequency at which XXY females were generated (binomial test: *P* = 0.86), and so we combined the datasets in a single maximum likelihood model (S1 Text; see Table 1 for parameter estimates). We found that the rate of sex chromosome nondisjunction in females was 1.49 × 10^–3^, similar to previous reports [14], and that the corresponding rate in males was significantly lower (5.79 × 10^–4^; LRT: χ^2^ = 4.22, df = 1, *P* = 0.04). Considering nondisjunction in both parents, our measurements indicate that the XXY karyotype will arise *de novo* in one out of every 776 females; this rate is statistically indistinguishable from estimates from 32 wild-derived strains [35] (Wilcoxon rank sum test, *P* = 0.69), suggesting that the population we studied has a representative rate of nondisjunction despite its history of long-term lab maintenance. Nondisjunction will produce both XXY and X∅ progeny, but the latter karyotype can also arise due to the spontaneous loss of a chromosome that disjoined normally [14,37–41]. We found a significantly higher rate of chromosome “loss” in female gametes than in male gametes (LRT: χ^2^ = 29.50, df = 1, *P* = 5.58 × 10^–8^).

**Table 1:** Maximum likelihood model results concerning sex-specific rates of non-disjunction (rate µ) and chromosome loss (rate *λ*).

Parameter	Estimate	SE	95% CI	*z*	*P*
*µ*_female_	1.49 × 10^–3^	4.24 × 10^–4^	(0.79, 2.44) × 10^–3^	3.52	4.32 × 10^–4^
*µ*_male_	5.79 × 10^–4^	1.94 × 10^–4^	(2.67, 9.39) × 10^–4^	2.98	2.89 × 10^–3^
*λ*_female_	6.30 × 10^–3^	1.26 × 10^–3^	(4.00, 8.97) × 10^–3^	5.00	5.70 × 10^–7^
*λ*_male_	5.50 × 10^–5^	3.08 × 10^–4^	(0.00, 7.29) × 10^–4^	0.18	0.86

XXY females that appear *de novo* inherit a Y chromosome from their male parent, whereas XXY females derived from existing XXY females will inherit a Y chromosome from their female parent (assuming no further nondisjunction). Additionally, XXY females derived from existing XXY females could be subject to a maternal effect of the XXY karyotype. We performed additional experiments, described below, to assess whether maternal karyotype and Y chromosome parent of origin influenced female phenotypes.

### Meiotic products of aneuploid flies

The persistence of supernumerary Y chromosomes could depend on the types of offspring produced by aneuploid flies. Previous studies indicate that the likelihood of non-disjunction can be increased in flies with existing sex chromosome aneuploidy, termed “secondary” nondisjunction [14,15] (in XXY females: XX ⟷ Y; in XYY males: X ⟷ YY); we conducted several crosses that allow such events to be phenotypically detected. We did not observe cases of secondary non-disjunction in either XXY females (S4-S6 Figs and S3-S5 Tables) or XYY males (S7 Fig and S6 Table). Applying the Clopper-Pearson confidence interval procedure [42] and the nondisjunction rate estimator of Cooper [35,43], using our aggregate data the upper 95% confidence limit for the rate of secondary nondisjunction in females is 2.16 × 10^–3^, statistically indistinguishable from our estimate of the “primary” nondisjunction rate (above and Table 1). This result could arise as an artifact of spontaneous alterations to chromosome marking, but such issues would not be consistent with other patterns we observed (S2 Text). A low rate of secondary nondisjunction means that XXY females will rarely produce inviable offspring karyotypes (i.e., XXX and YY) when mating with normal males.

### Viability effects of phenotypic markers and sex chromosome aneuploidy

**Fig 1:**
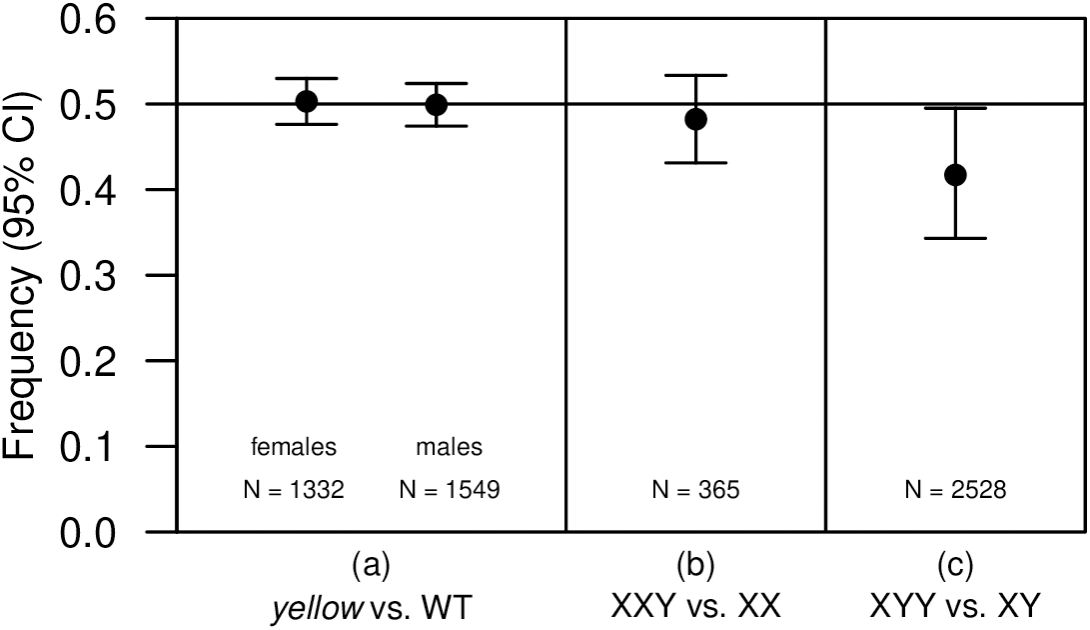
Viability effects of markers and aneuploidy. In the absence of a viability effect, we expect a frequency of 0.5 in each case. Sample sizes (N) reflect the total numbers of flies scored. (A) The phenotypic marker used to indicate aneuploidy (*yellow*) did not significantly affect viability in either sex (GLM; females: *df* = 29, *z* = 0.219*, P* = 0.826; males: *df* = 29, *z* = –0.076, *P* = 0.939; S7 Table). (B) The viability of XXY females did not differ significantly from that of XX females (GLM; *df* = 2, *z* = –0.68, *P* = 0.496; S8 Table). (C) We find evidence that the viability of XYY males is reduced relative to that of XY males (GLMM, *z* = –3.539, *P* = 4.01 × 10^–4^; see S3 and S4 Tables).

For our fitness assays we used body color to distinguish XX females (yellow-bodied) from XXY females (normal color due to a *y*^+^ allele marking the Y chromosome). Using a control cross with euploid flies, we found no evidence that the *yellow* phenotype was associated with reduced viability in either females or males (Fig 1a and S7 Table), indicating that our measures of aneuploid viability were not biased by marker effects. We found that XXY females did not have reduced viability relative to XX females (Fig 1b and S8 Table). In contrast, we found that XYY males had reduced viability relative to XY males (Fig 1c and S3 and S4 Tables). We can calculate a viability selection coefficient for the XYY karyotype as *s* = (1 – 2*f*)/(1 – *f*), where *f* is the frequency of XYY males out of the total number of males; our best estimate of this value is *s* = 0.21 (95% CI: 0.10–0.31).

A smaller experiment involving the compound chromosome C(1;Y)2, *y*^1^ *B*^1^ (S6 Fig and S5 Table; 1286 offspring) showed a non-significant effect of the X∅ karyotype on viability relative to XY (*s* = –0.11, 95% CI: –0.30 to 0.05, *P* = 0.19), and a non-significant effect of the XXYY karyotype on viability relative to XXY (*s* = 0.12, 95% CI: –0.03 to 0.25, *P* = 0.11); recall that X∅ males are entirely sterile; XXYY females are reported to have reduced fertility [14].

### Female reproductive traits and mass

**Fig 2:**
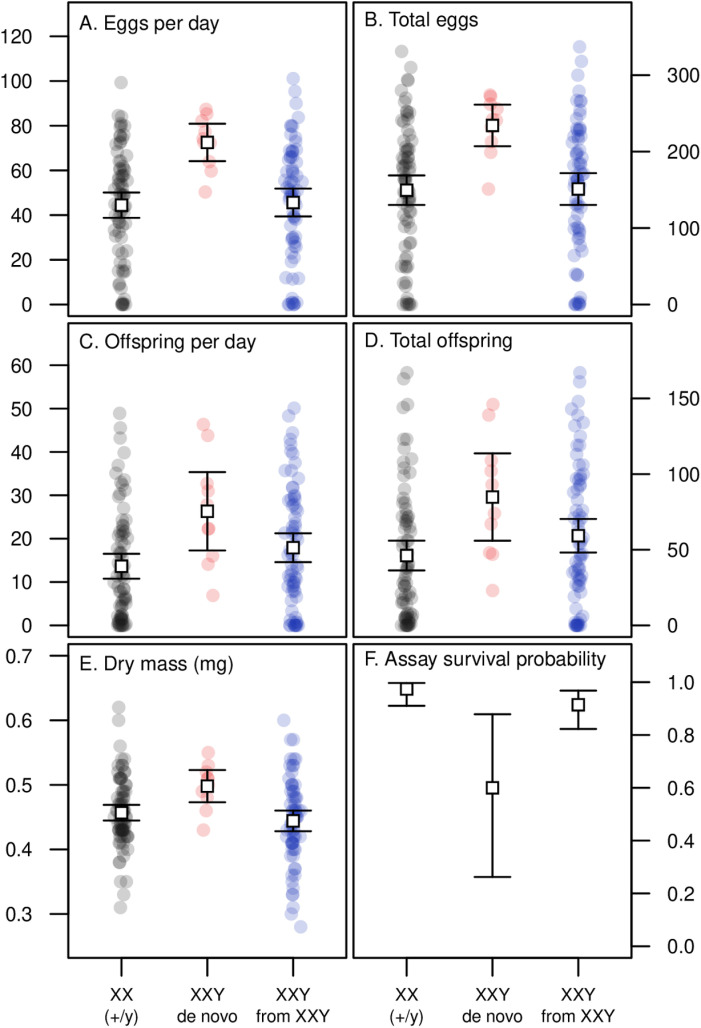
Adult productivity, mass and survivorship of XXY and XX females. Error bars represent 95% confidence intervals. Eggs per day (A) and offspring per day (C) reflect productivity averaged over the number of days a female survived during the assay, whereas total numbers (B and D) reflect productivity regardless of longevity. The results of Kruskal-Wallis tests for variation among groups for each trait are as follows. (A) χ^2^ = 12.683, *df* = 2, *P* = 1.761 × 10^–3^. (B) χ^2^ = 11.002, *df* = 2, *P* = 4.083 × 10^–3^. (C) χ^2^ = 9.04, *df* = 2, *P* = 0.0109. (D) χ^2^ = 8.155, *df* = 2, *P* = 0.0170. (E) χ^2^ = 8.696, *df* = 2, *P* = 0.0129. (F) χ^2^ = 17.757, *df* = 2, *P* = 1.393 × 10^–4^. In each case, pairwise tests (Wilcoxon rank sum tests) indicate that XXY females derived from spontaneous non-disjunction events (*de novo*) differ from the other groups. Compared with XX females, spontaneous XXY females produced approximately 63% more eggs per day (*P* = 4.465 × 10^–4^), 57% more eggs in total (*P* = 1.217 × 10^–3^), 93% more offspring per day (*P* = 4.773 × 10^–3^), 84% more offspring in total (*P* = 7.569 × 10^–3^), had 9% greater dry body mass (*P* = 9.463 × 10^–3^), and had a 38% lower chance of surviving to the end of the 4-day assay (*P* = 1.562 × 10^–5^). For XXY females from stock there was also a non-significant trend towards elevated offspring production compared with XX females (per day: *P* = 0.079; total: *P* = 0.100), and reduced survival (*P* = 0.0966). See S9 Table.

**Fig 3:**
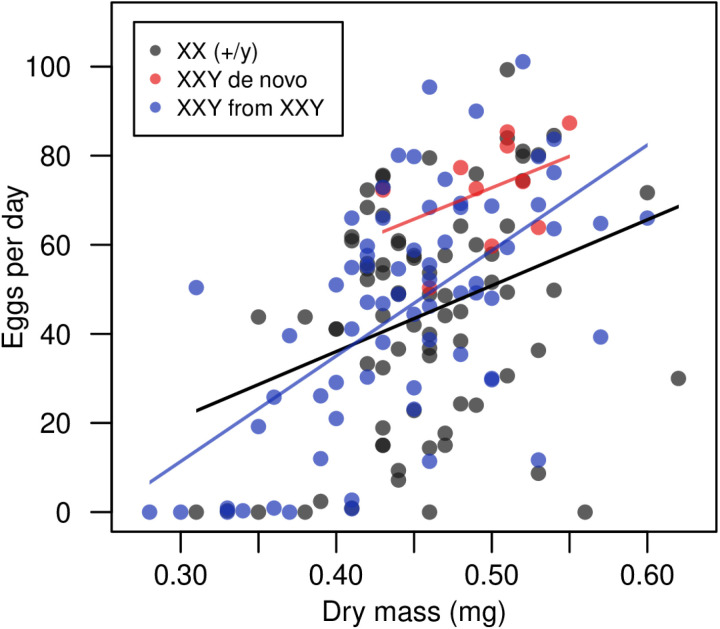
Egg production and body mass in XXY and XX females. Linear regression lines are shown for each group. A positive correlation between body mass and egg production is apparent for all three types of females (ANOVA comparing nested models; no evidence of interaction between treatment and mass: *F* = 1.080, *df* = 2, *P* = 0.342; effect of mass: *F* = 42.567, *P* = 9.447 × 10^–10^). See S9 Table.

We examined the reproductive traits of XXY females that were derived from spontaneous non-disjunction events (S1 Fig) as well as from pre-existing XXY females (S2 Fig), as compared with XX females (S9 Table). We assessed female attractiveness in mate choice trials with one focal female (XX or XXY), one standard female (euploid and brown-eyed), and one wild type male. Out of 190 attractiveness trials, we observed mating in 157 cases. We found no evidence that karyotype or parent of origin affected whether the focal female was the first to mate (chi-squared test, χ^2^ = 0.639, df = 2, *P* = 0.727), or the time to the first mating (Kruskal-Wallis test, χ^2^ = 0.837, df = 2, *P* = 0.658). However, we found evidence for significant variation among groups in traits related to offspring production (Fig 2A-D). In each case, XXY females derived from spontaneous non-disjunction events displayed greater reproductive output than XX females or XXY females derived from an XXY stock and were also larger in terms of dry body mass (Fig 2E). Such females were also less likely to survive until the end of the assay (Fig 2F), but this reduced longevity did not completely offset their greater fecundity over this period. For individuals where we obtained both fecundity and mass data, these traits were positively correlated for all three female types (Fig 3). The greater mass of spontaneous XXY females relative to stock XXY females was found for both females that survived for the whole fecundity assay (N = 70, Wilcoxon rank sum test, *P* = 0.049) and those that did not (N = 9, Wilcoxon rank sum test, *P* = 0.016).

The above assays compared XX and XXY females that were reared apart, allowing us to compare flies with the same marker phenotype (non-yellow), but requiring development in separate vials. We repeated our assay of body mass using XX and XXY females reared together in the same vials, which were distinguishable by marker phenotype, and where the maternal parent was always XXY (S2 Fig and S10 Table). In this case the XXY females showed 16.6% greater average body mass than the XX females (*t* = 3.563, df = 58, *P* = 7.422 × 10^–4^), as compared with a 9% difference for flies reared separately (Fig 2). However, this difference could be driven by marker effects on body mass.

### Egg size

**Fig 4:**
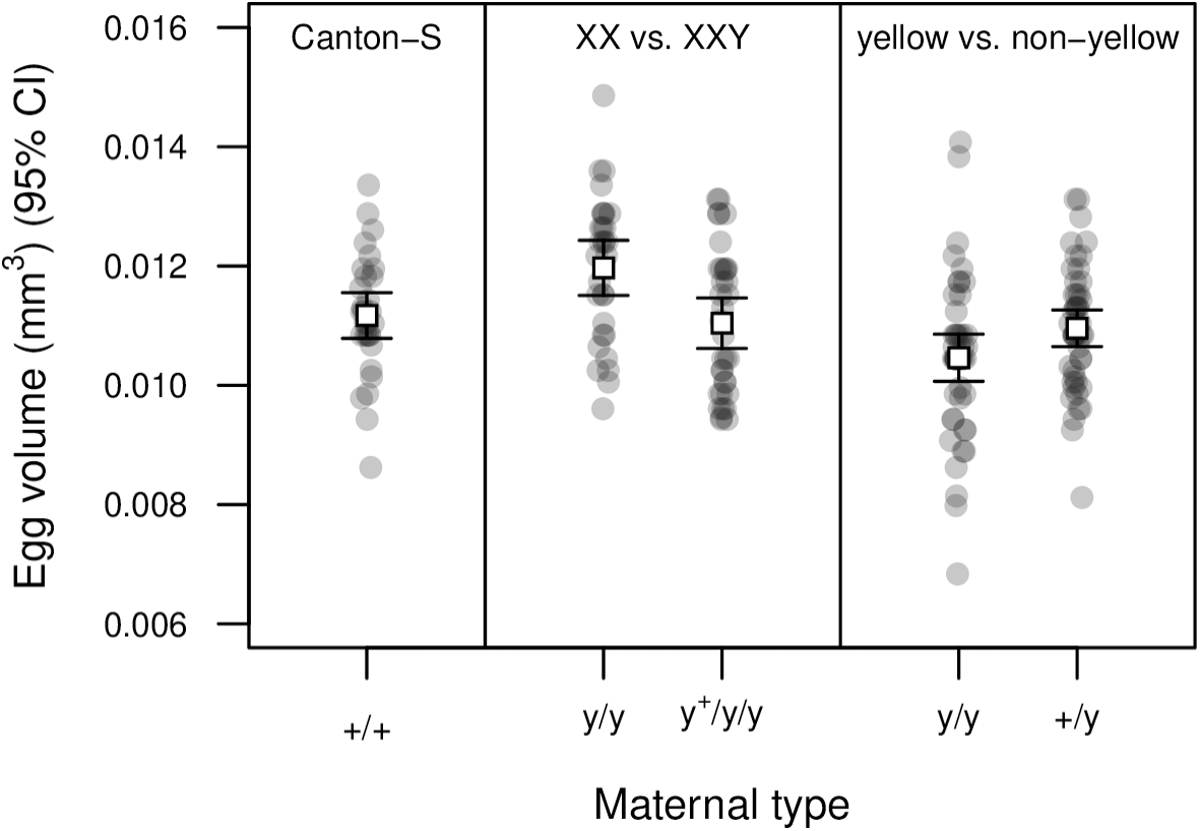
Egg sizes of XXY and XX females. Egg sizes for wild-type Canton-S flies are shown for comparison (left; *N* = 30). XXY females produced eggs that were 7.7% smaller, on average, than those of XX females reared in the same vials (center; *t* = 3.024, *df* = 62, *P* = 3.63 × 10^–3^). This is not likely the result of differences in marker phenotype, since XX females expressing the *yellow* phenotype produced eggs that were 4.72% smaller than those of non-*yellow* XX females (*t* = 1.987, *df* = 92, *P* = 0.0499. See S11 Table.

Given that XXY females produced more eggs (Fig 2), we asked whether their eggs were also smaller. We first reared XX and XXY females in the same vials (distinguishable by marker phenotype; derived from XXY parents), collected eggs from cage populations that included males, and measured eggs individually using a microscope. We found that the XXY females produced smaller eggs on average (Fig 4 and S11 Table). This pattern could arise if the *yellow* phenotype was associated with larger eggs (here the XX females expressed the *yellow* phenotype and XXY females did not), but a comparison of egg sizes for *yellow* and non-*yellow* XX females showed that, if anything, the yellow marker was associated with smaller eggs (Fig 4), suggesting that reduced egg size was truly associated with the XXY karyotype.

### Development time

**Fig 5:**
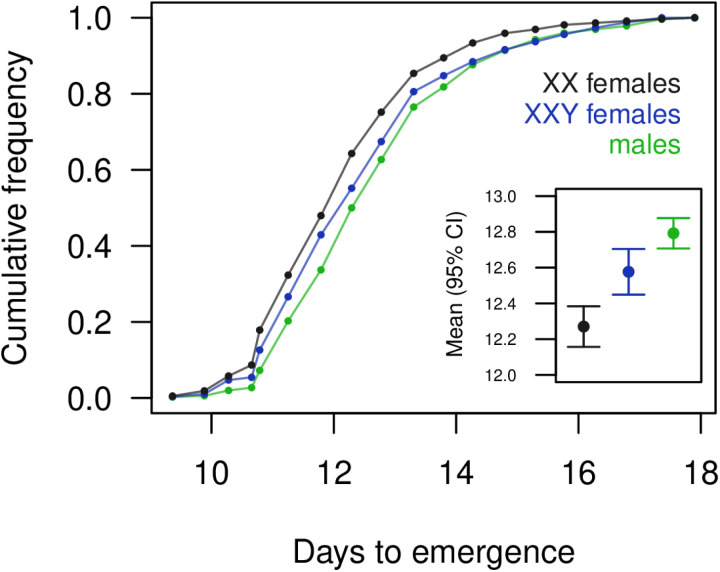
Development time in XXY and XX females, and males. The cumulative frequency of emerging XX females (*N* = 588) increased more rapidly than that of XXY females (*N* = 571). Males (which generally emerge later than females) are also shown for comparison (*N* = 1214) and will be a mix of XY and XYY. Inset: mean emergence time in days; emergence time was 7.3 hours later in XXY females than in XX females, on average (*t* = 3.525, *df* = 1157, *P* = 4.395 × 10^–4^). See S12 Table.

We reared XX and XXY females in the same vials (distinguishable by marker phenotype; derived from XXY parents) and found that the XXY females developed more slowly than the XX females (Fig 5 and S12 Table). This difference was significant but small, amounting to a 2.5% increase in development time for the XXY karyotype. We can’t exclude the possibility of a marker effect where flies expressing the *yellow* phenotype (XX in this case) develop more quickly, but we consider this explanation unlikely. The overall frequency of XXY females in this assay did not differ from 50% of females (binomial test, *N* = 1159, *P* = 0.638), reinforcing our inference above that viability selection against the XXY karyotype was minimal.

### Maternal effects

**Fig 6:**
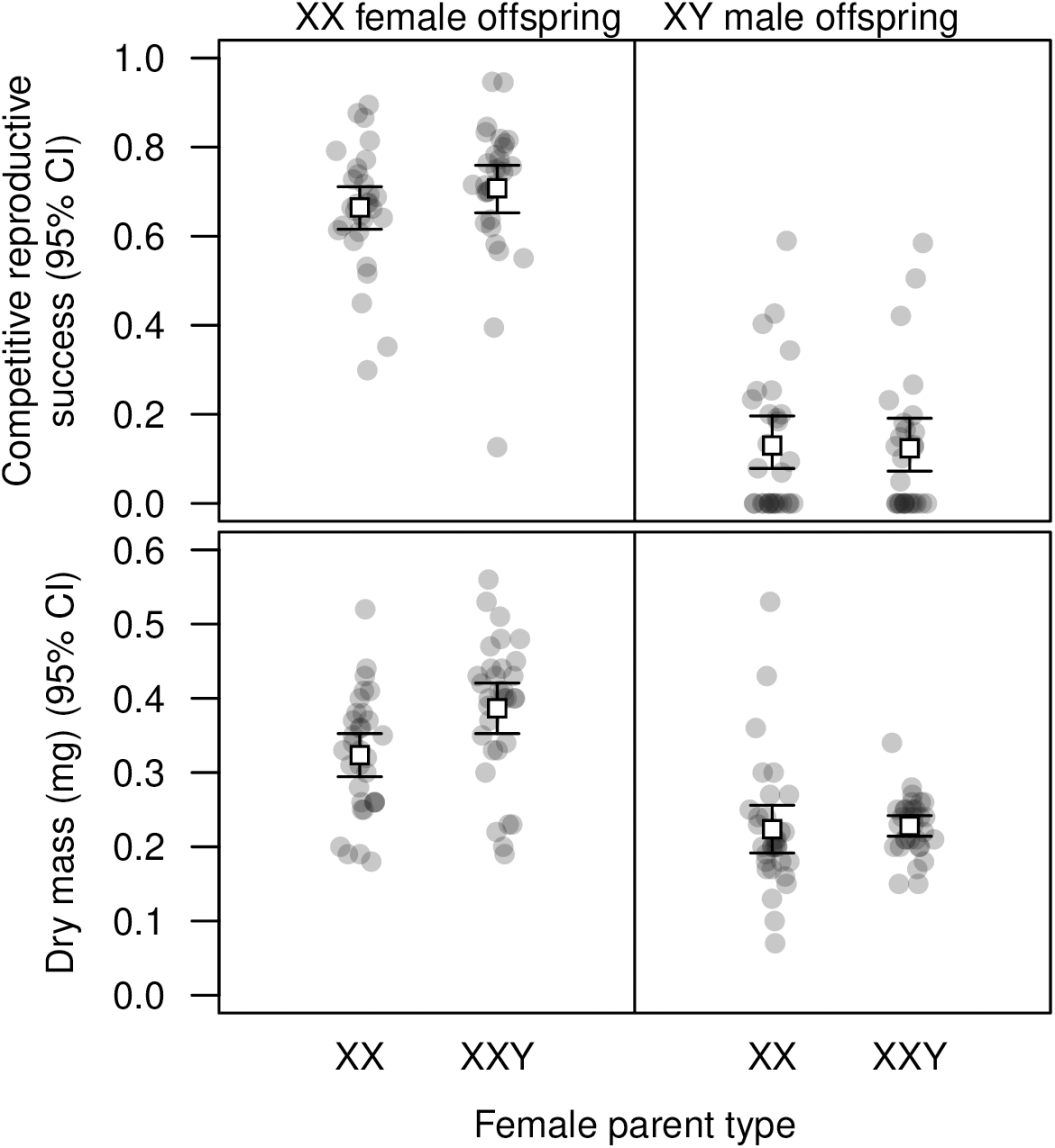
Maternal effects of the XXY karyotype on XX and XY offspring. Open squares represent means. Maternal karyotype did not significantly affect the reproductive success of XX female offspring (top left; *N* = 60 vials, 6882 flies; quasibinomial regression: *t* = 1.175, *P* = 0.245), or XY male offspring (top right; *N* = 59 vials, 6289 flies; quasibinomial regression: *t* = –0.145, *P* = 0.885; see S13 Table). Note that in this experiment, reproductive success was measured in flies expressing the *yellow* marker (see S9 Fig), which likely explains the low overall reproductive success in focal males relative to competitors (top right). Maternal karyotype significantly affected the body mass of XX female offspring (bottom left): XX offspring of XXY parents were 19.5% larger on average than XX offspring of XX parents (t-test: *t* = –2.877, *df* = 62, *P* = 5.493 × 10^–3^). This was not the case for XY male offspring (bottom right; t-test: *t* = –0.255, *df* = 62, *P* = 0.799; see S14 Table).

Our data on fecundity-related traits and body mass (see above; Fig 2) suggested that XXY females derived from spontaneous non-disjunction events had higher trait values than XXY females derived from pre-existing XXY females. To assess whether this could be due to a negative maternal effect of the XXY karyotype, we compared the traits of XX and XY offspring derived from XX versus XXY females. In this assay, the flies of interest had the same marker phenotype within each sex but were reared separately. We found that maternal karyotype did not significantly affect offspring reproductive success, but did affect the body mass of female offspring, with XXY parents producing larger XX offspring than XX parents (Fig 6 and S13 and S14 Tables). This indicates that, if anything, the maternal effect of the XXY karyotype on these traits is positive.

### XYY male reproductive success

**Fig 7:**
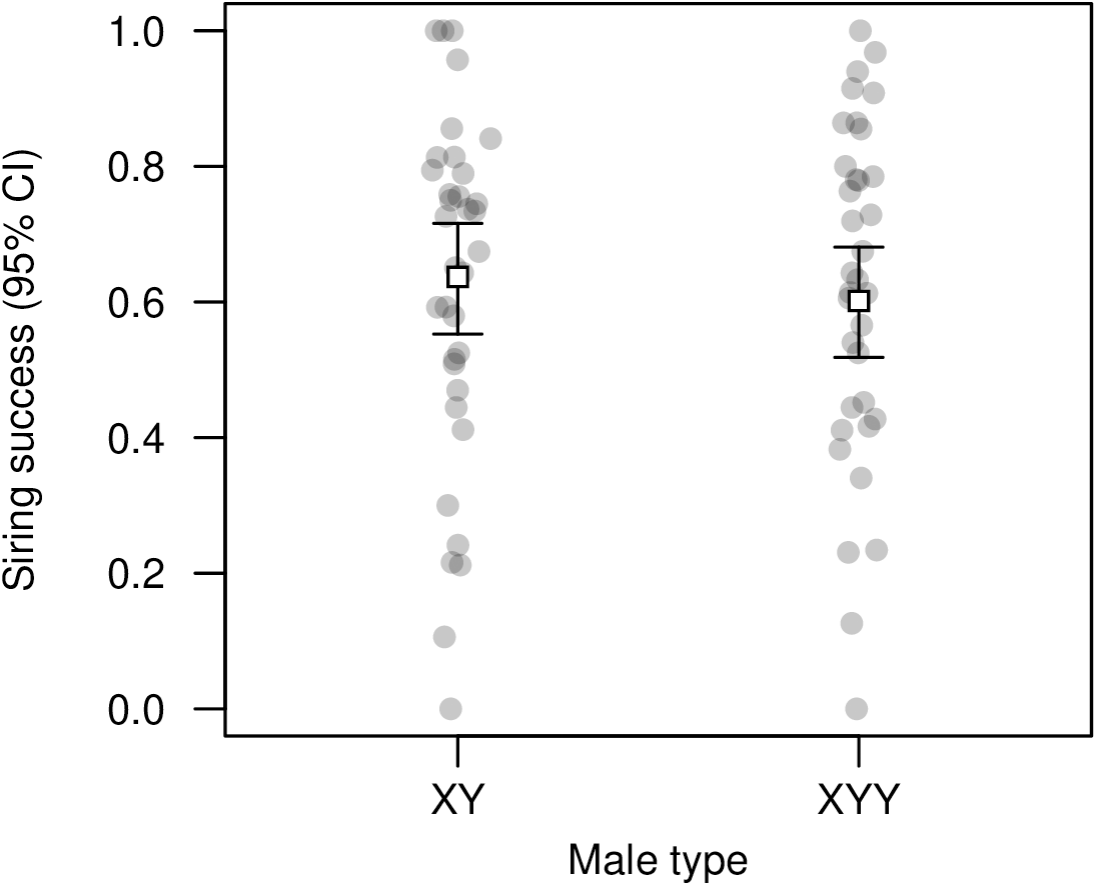
Adult reproductive success of XY versus XYY males. Open squares represent means. Focal males (XY or XYY, reared separately) competed with *bw*/*bw* males to reproduce with *bw*/*bw* females, where *bw* is a recessive eye color marker. The plot shows the proportion of offspring with the wild-type eye color in each vial, representing a measure of the siring success of focal males. N = 35 vials per treatment, with 6144 offspring scored in total. We find no evidence that siring success differs between these karyotypes (quasibinomial GLM: *t* = 0.599, *P* = 0.551; see S15 Table).

In competition with phenotypically marked (brown-eyed) males, focal XY males sired 63.7% of offspring on average, whereas focal XYY males sired 60.1% of offspring on average, a non-significant difference (Fig 7 and S15 Table). This indicates that the XYY karyotype does not affect male reproductive success.

### Frequency change

**Fig 8:**
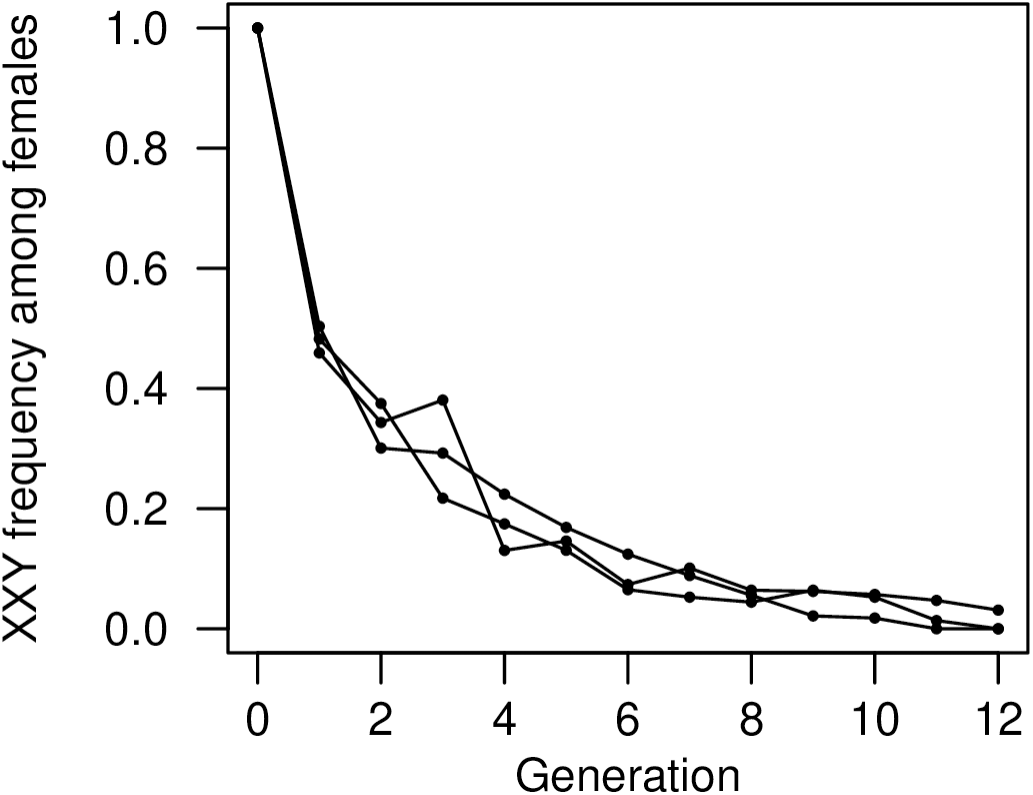
Frequency of XXY females over time in experimental populations. Among females, the frequency of the XXY karyotype declined significantly over the course of 12 generations (quasibinomial GLM: *t* = –11.87, *P* = 3.53 × 10^–14^; see S8 Table). Each line represents an independent population with 287 adults per generation on average.

As an alternative to measuring specific traits, we used experimental evolution to examine the change in the frequency of XXY females over time, which will reflect overall selection on XXY females, XYY males, and additional karyotypes created through XXY-XYY matings (e.g., XXYY). In populations initially composed entirely of XXY females, the XXY frequency dropped to about 0.5 after one round of reproduction (Fig 8 and S8 Table), reflecting the fact that only half of the female offspring of XXY females will be XXY (S2 Fig), and that the XXY karyotype does not have reduced viability (Fig 1). In subsequent generations, the XXY frequency continued to decline (Fig 8 and S8 Table); in the absence of any selection this continued decline is not expected, since XXY females will be generated by XYY sires, i.e., there would be no net change in the frequency of supernumerary Y chromosomes. We explored this pattern using stochastic simulations (10000 per scenario). We first asked whether the observed rate of frequency decline could occur solely due to selection against complex karyotypes like XXX and XXYY, i.e., in the absence of any direct fitness effect of the XXY or XYY karyotypes. We simulated populations of the same size as the experimental populations, with the same starting composition, non-overlapping generations, random mating, and non-disjunction at the rates determined above. In these simulations we assume that the karyotypes X∅, XXX, XXYY, etc., have zero fitness. In the absence of any fitness effects of the XXY or XYY karyotypes, we found that the simulated frequency change was rarely as negative our observed data (one-tailed *P* = 0.019), and that the average XXY frequency after 12 generations of evolution was rarely as low as that of our observed data (one-tailed *P* < 1 × 10^–4^). This result indicates that some selection on XXY or XYY is necessary to explain the observed data. While the decline in XXY frequency could reflect an impact of this karyotype on an un-measured female fitness component, we considered whether the viability selection against XYY males described above is sufficient to explain this pattern. Using the point estimate *s*_XYY_ = 0.21, 9.4% of simulated populations displayed a rate of XXY frequency change at least as extreme as the observed rate. Using the lower 95% confidence limit of *s*_XYY_ = 0.10, 4.3% of simulated populations show a result this extreme; using the upper 95% confidence limit of *s*_XYY_ = 0.31, 16.4% of simulated populations show a result this extreme. We therefore conclude that viability selection against XYY males can plausibly explain the observed decline in XXY frequency in our experimental populations, even in the absence of direct selection against XXY females.

### Standing frequency

We used two methods to estimate the standing frequency of the XXY karyotype in a laboratory population. Using the test cross method, we detected one XXY female out of 158 tested (S16 Table). Using the PCR method, we detected one XXY female out of 75 tested. These rates are not statistically distinct (Fisher’s exact test, *P* = 0.54), so we proceeded to analyze these data in combination using Approximate Bayesian Computation (ABC), accounting for detection bias in the test cross method (see Materials and Methods). Based on 50000 posterior values, we estimate a standing frequency of the XXY karyotype among females of 0.0133 (95% CI: 0.0027–0.0320). In other words, our best estimate is that about one in every 75 females in this laboratory population has the XXY karyotype (95% CI: 1/31–1/370).

### Estimating fitness effects of XXY based on mutation-selection balance

As an alternative means of estimating the fitness of the XXY karyotype (*w*_XXY_), we considered a model of mutation-selection balance, where the expected standing frequency of a variant at equilibrium, q*, is determined by the rate of mutation, µ, and the strength of purifying selection, *s*, where q* ≅ µ/s. We sought to determine the value of *w*_XXY_ that would produce the observed standing frequency of XXY (*f*_XXY_), given the sex-specific rates of mutation (nondisjunction) we observed. We generated a deterministic model of karyotype frequency change (S3 Text), taking into account the observed viability of the XYY karyotype (*w*_XYY_), and solved for two unknown parameters: *w*_XXY_ and the standing frequency of XYY males (*f*_XYY_). We obtained credible intervals for unknowns by applying the model with values for *f*_XXY_ and *w*_XYY_ sampled from their respective posterior distributions. Based on this model, we estimate that XXY females have fitness *w*_XXY_ = 1.009 (95% CI: 0.708–1.110), meaning that we cannot reject the null hypothesis that XXY females have the same reproductive success as XX females. This model also indicates that about one in every 200 males will have the XYY karyotype at equilibrium (*f*_XYY_ = 0.005; 95% CI: 0.001–0.012).

The model results above assume that nondisjunction rates are not influenced by karyotype, but we also explored the impact that elevated nondisjunction in aneuploid flies (secondary nondisjunction) would have on these inferences. If we assume a rate of secondary nondisjunction of 0.032 in both sexes [14], the model results for *w*_XXY_ are only slightly different (*w*_XXY_ = 1.024, 95% CI: 0.717–1.126), and the results for *f*_XYY_ are essentially unchanged. In general, the model shows that higher secondary nondisjunction rates are associated with higher estimated values of XXY female fitness, with *w*_XXY_ > 1.

## Discussion

Our goal was to quantify the impact of sex-chromosome nondisjunction at the population level. We first measured how often nondisjunction occurs in each sex. Our data (Table 1) are consistent with previous rate estimates, but clarify that female parents are the main source of both nondisjunction events (72%) and chromosome losses (99%). In *Drosophila* and other species, there are sex differences in key aspects of meiosis, which could translate into different realized rates of nondisjunction [44–47]. In terms of its genetic consequences, female-biased nondisjunction implies that most XXY flies that arise *de novo* will be X_M_X_M_Y_P_ rather than X_M_X_P_Y_P_, where M and P refer to chromosomes of maternal versus paternal origin, respectively. Most human aneuploidies are also maternally derived, though male and female parents contribute similarly to the occurrence of sex chromosome nondisjunction underlying Klinefelter syndrome (XXY) [6,7].

We next considered fitness components in aneuploid flies. The XXY karyotype appears by nondisjunction, but the XYY karyotype typically does not (assuming XX and XY parents). However, roughly half of the male offspring of XXY females will be XYY, and so the fitness of both types is relevant. We found evidence for reduced viability in XYY males, but not in XXY females (Fig 1). For XYY, our data show a selection coefficient of *s* = 0.21 (95% CI: 0.10–0.31), which is substantial and consistent with early reports [48,49]. A false signal of viability selection against XYY in our assays could arise if XXY parents preferentially transmit X gametes over XY gametes (see S4 and S5 Figs), but the fact that we recovered XX and XXY females equally in these assays indicates that this is not the case. Sex-specific viability selection against deleterious alleles in flies may not be uncommon [50], though this case appears to represent an extreme example. Further quantification of viability in Y-chromosome aneuploids would be valuable in addressing whether these effects are due to the copy number of the Y chromosome as a whole versus specific elements.

While we found that XX and XXY females did not differ in viability, our experiments revealed differences in several other traits. We had the opportunity to examine two types of XXY females: those that appeared *de novo* by nondisjunction and those that were derived from pre-existing XXY females. We found that XXY females arising *de novo* had greater body mass and early-life fecundity than either XX females or XXY females derived from XXY parents (Fig 2), though mass was positively correlated with fecundity in all groups (Fig 3), as expected [51]. In principle, this difference could arise if the XXY karyotype conferred increased mass and fecundity but also had a negative maternal effect. In additional experiments, we found no evidence for a negative maternal effect of the XXY karyotype; in fact, we detected a positive maternal effect of XXY on the body mass of XX offspring (Fig 6). An alternative explanation is that the parent of origin of the Y chromosome is relevant to XXY female fitness: XXY flies produced through spontaneous nondisjunction have a paternally derived Y chromosome, whereas XXY flies derived from existing XXY flies will receive their Y chromosome from their female parent, as long as no additional nondisjunction has taken place (S2 Fig).

A parent-of-origin effect could arise through imprinting, an epigenetic process where heritable chromosome marks are established in a sex-specific fashion [52]. Genome-wide imprinting in flies has been studied in detail, with mixed results as to its prevalence [53–56], but there is consistent evidence for imprinting on the Y chromosome [26,33,34,57,58]. Our results suggest that male-imprinted Y chromosomes increase the size and fecundity of XXY females, representing a positive but transient outcome of sex chromosome nondisjunction; XXY female viability could also be affected by parent of origin, but our assays do not address that possibility. Surprisingly, the increased size of *de novo* XXY females did not confer a detectable increase in attractiveness in mate preference trials, which would be expected [51], but we may simply lack the power to detect such a relationship in a small sample. We recommend that future studies of aneuploid flies consider the possible influence of parent of origin effects.

While we found that *de novo* XXY females produced eggs and offspring at an elevated rate, they also showed reduced survivorship during our assays (Fig 2F), which would counteract any fecundity benefits of this karyotype. In contrast, XXY females derived from XXY parents showed slightly elevated fecundity and slightly reduced survival relative to XX females (Fig 2; these differences are marginally nonsignificant). This suggests that the XXY karyotype has neutral to positive effects on fecundity, and neutral to negative effects on longevity, depending on the Y chromosome parent of origin. Previous research (conducted with compound chromosomes) has also indicated that XXY females have reduced lifespan [25], but those findings may have been driven by confounding effects of genetic background [29].

The effects of Y chromosomes on female fecundity led us to examine whether the eggs produced by XXY flies differ from those of XX flies. We found that XXY females produced eggs that were 7.7% smaller than those of XX females reared in the same vials (Fig 4). There is evidence that gene expression in ovaries is particularly sensitive to the presence of a Y chromosome [30], and the nucleotide content of unfertilized eggs differs between XX and XXY females [59,60]. Egg size is known to be positively correlated with embryonic viability and the rate of larval development [61], so the production of smaller eggs could represent a fitness cost for the XXY karyotype.

We detected another potential cost of the XXY karyotype in the form of delayed development, with the presence of a Y chromosome delaying adult female emergence by about seven hours, on average (Fig 5). This could slow the spread of supernumerary Y chromosomes through a population but could also permit XXY females to reach a larger adult size (Fig 2E). We did not directly test for an effect of supernumerary Y chromosomes on male development time, as XY and XYY males cannot be distinguished in this assay. However, we note that males emerged 12.5 hours later than XX females, on average, which is larger than the typical level of sexual dimorphism for this trait, four hours [62]. If we assume that XY males take four hours longer to develop than XX females, and account for the reduced frequency of XYY males due to viability selection, our data imply that an extra Y chromosome in males adds 19.3 hours to development time; considering the range of sexual dimorphism observed [62], we would infer that an extra Y chromosome in males adds 15.6–28.4 hours to development time. We therefore see suggestive evidence that supernumerary Y chromosomes delay development in both sexes, with a stronger delay in males, but this inference is indirect in the case of males. Further work could clarify the effect on males and establish the specific stages of development responsible for these effects.

Several prior studies indicate that variance in male reproductive success is a major source of selection against deleterious mutations in flies [63–72], and we considered whether this could also be the case for sex chromosome aneuploidy. There is evidence that reduced male fertility can occur in the presence of supernumerary Y chromosomes, particularly in the presence of three or more [20–24], but we did not find a difference in realized reproductive success between XY and XYY males (Fig 7). This discrepancy could stem partly from the use of different marking schemes: prior studies use compound chromosomes, with males hyperploid for duplicated regions containing phenotypic markers, whereas we studied males with two free Y chromosomes, only one of which was marked with Dp(1;Y)*y*^+^. Another possible explanation is that patterns of sexual selection differ depending on whether copulation success is considered versus realized reproductive success in the context of both pre- and post-copulatory competition [73,74]. Strong reproductive success in XYY males will help maintain supernumerary Y frequency since, in the absence of additional nondisjunction, all of their female offspring will be XXY.

We are interested in understanding total selection on supernumerary Y chromosomes; of the traits we examined, viability selection against XYY males is the largest contributor, but there is also evidence for subtler effects on other traits, such as female fecundity and longevity. Predicting overall selection based on these traits is difficult because their relative importance for total fitness is unknown, and we can’t exclude the possibility of selection via unmeasured traits. In an effort to understand overall selection regardless of the specific traits involved, we took two additional approaches based on experimental evolution and mutation-selection balance.

In replicate populations that were initiated with XXY females and XY males and allowed to evolve for 12 generations, we saw rapid and consistent declines in the frequency of the XXY karyotype (Fig 8). Using simulations, we established that viability selection against XYY males (Fig 1) is sufficient to explain the observed rate of frequency decline. Additionally, in this artificial scenario with an initially high frequency of supernumerary Y chromosomes, mating between aneuploid males and aneuploid females will not be uncommon, which will give rise to XXYY females and other hyperploid karyotypes that have reduced viability and fertility [14]. These experimental evolution data are therefore consistent with the idea that XXY females have total fitness similar to XX females.

Another approach to understanding total selection on the XXY karyotype relies on the concept of mutation-selection balance, where the standing frequency of a mutant type reflects a balance between its appearance by nondisjunction and its removal by selection. Using both PCR and test crosses, we found that the standing frequency of XXY among females in an un-marked laboratory population was approximately 1/75. This is about ten times higher than the frequency of spontaneous XXY flies appearing *de novo*, and so a crude estimate of net selection against supernumerary Y chromosomes is 10%, consistent with the observed viability selection of 21% against half of the affected individuals (XYY males). We developed a formal model of these dynamics, accounting for the nature of sex chromosome transmission and uncertainty in our estimates of nondisjunction rates and standing frequency. This model indicates that, in the presence of viability selection against XYY males, the observed standing frequency of XXY females can emerge even without any direct selection for or against this karyotype (i.e., *w*_XXY_ ≅ 1). This conclusion is essentially unchanged when we assume a higher rate of secondary nondisjunction; indeed, to the extent that XXY females produce XX and Y gametes through secondary nondisjunction, progeny with little or no viability will often result (XXX and YY), such that XXY females must be more productive to explain a given XXY standing frequency. It would be valuable to apply this model to data on standing XXY frequency in populations believed to experience higher rates of secondary nondisjunction.

To summarize, we found that the XXY karyotype affected some specific traits, but these effects appeared to be relatively weak and mixed in direction. If there are fitness benefits to the XXY karyotype, these are most likely experienced by females that received a Y chromosome from their male parent. Experimental evolution of the XXY frequency and the standing XXY frequency under mutation-selection balance are also consistent with XXY females having near-wild-type fitness, given the observed level of viability selection against XYY males. It therefore appears that, while the presence of a Y chromosome may substantially alter female transcription patterns [26–30], affected individuals are relatively robust to these changes in terms of fitness. However, we cannot exclude the possibility that the effects of aneuploidy in natural populations could differ from those detected in the lab, particularly given the long-term maintenance of Canton-S in small population sizes. In a wild strain of *D. melanogaster* isolated from the Seychelles in 1987, the X-linked rDNA locus is greatly reduced, and females of this strain each carry one or two Y chromosomes as a source of rDNA [31]. These authors also found evidence that the supernumerary Y chromosome subsequently evolved to become truncated, potentially reducing costs for females but also not supporting male fertility; however, the original order of events and selective pressures in this case are not clear [31]. Our data suggest that the XXY karyotype may not be especially harmful to females and is not very rare in the laboratory strain we studied; if these patterns hold true for wild populations, it could allow the loss of X-linked rDNA to be tolerated, as in this example, and potentiate karyotype evolution more generally.

## Materials and methods

### Statistical analyses

We conducted statistical analyses and prepared figures in *R* [75] (S1 File). Specific analysis approaches are noted throughout the text. We applied non-parametric methods when non-normality was indicated. We analyzed binomial data using generalized linear models (GLM), accounting for overdispersion if present, and including random effects terms in some cases using the *lme4* package (GLMM) [76].

### Fly culturing

We performed all experiments using flies reared on defined yeast-sugar-agar medium at 25C, under a 12:12 light:dark cycle. Unless otherwise noted, we conducted crosses under CO_2_ anesthesia using flies that were 2–5 days old post eclosion, with females collected as virgins, in standard vials seeded with live yeast.

### Genetic background and markers

We obtained the Canton-S wild-type genetic background from the Bloomington Drosophila Stock Center (RRID:BDSC_64349) in 2019 and maintained this strain as a large, outbred laboratory population. We used flies from this genetic background for our experiments, crossing phenotypic markers or marked chromosomes into this genetic background. To track Y chromosomes in crosses, we obtained flies with a phenotypically marked Y chromosome (RRID:BDSC_1531), which consists of the interchromosomal duplication Dp(1;Y)*y*^+^. In this strain, a distal section of the X chromosome including the gene *yellow* (*y*) is duplicated onto the tip of the Y chromosome. In the presence of a non-functional *y* allele on the X chromosome(s) (*y*^1^ in this case), flies carrying this marked Y chromosome have a wild-type body color, due to *y*^+^, and flies without a Y chromosome have a yellow body color. To estimate sex-specific rates of non-disjunction, we also used a similar stock where the Y chromosome is marked with the phenotypically dominant marker *Bar*^*S*^ (RRID:BDSC_1542). For additional tests, we also used a compound X-Y chromosome stock (RRID:BDSC_2487), and visible markers (*m*^*D*^, X-linked, RRID:BDSC_70; *bw*^1^, autosomal, RRID:BDSC_264), back-crossed into the Canton-S genetic background for 8 generations.

### Obtaining XXY females and XYY males

The XXY karyotype can arise due to spontaneous meiotic non-disjunction in females or males. We generated a large population of flies carrying the *y*^1^ allele on the X chromosome and the *y*^+^ allele on the Y chromosome (S1 Fig). Over the course of several generations, we identified non-yellow (XXY) females in this population and collected them as virgins. We used some XXY females to conduct further assays (see below) and others to create an ongoing stock (S2 Fig), from which we collected additional females for phenotypic assays. As part of our PCR-based assay of standing XXY frequency (see below), we confirmed the presence of Y-linked DNA in females from our XXY stock, as positive controls, and the absence of Y-linked DNA in XX females, as negative controls. Additionally, we used XXY females to generate XYY males for some assays (see below).

### Measuring spontaneous non-disjunction

During collection of XXY females (see above), we recorded numbers of XXY and XX flies to assess the spontaneous rate of non-disjunction. In this context, XXY females can be generated by non-disjunction in either parent (S1 Fig). When estimating the rate of non-disjunction, we disregarded the first two collection periods to exclude females that could have been the progeny of aneuploids present in the population before collection began. We wanted to additionally distinguish female from male non-disjunction, in part to better parameterize models of aneuploid frequency; to achieve this, we performed a test cross with a distinct marking scheme (S3 Fig) where the products of female and male non-disjunction have different phenotypes. The female parents in this assay were derived from a stock with a marked Y chromosome to ensure that they were XX. We used maximum likelihood optimization to determine the sex-specific rates of non-disjunction and chromosome loss [14,37–41] that would give rise to the observed data (S1 Text), applying the *R* package *bbmle* [77].

### Meiotic products of aneuploid flies

We performed several crosses to assess the meiotic products of both XXY females (S4-S6 Figs) and XYY males (S7 Fig). In the latter case, we first selected putative XYY males (bearing two marked Y chromosomes, S2 Fig) based on the “hairy-wing” phenotype, which is sensitive to Dp(1;Y)*y*^+^ dosage [14], and then verified the karyotype of these sires by examining offspring phenotypes (S7 Fig).

### Viability effects of phenotypic marker and sex chromosome aneuploidy

As our experiments often require identifying alternative karyotypes using the presence or absence of the *yellow* phenotype, we examined whether such marker variation itself affected viability in each sex with standard karyotypes (S8 Fig). We analyzed these data using generalized linear models; there were no indications of overdispersion. Finding no evidence for marker effects (see *Results*), we can use the phenotype frequencies in certain crosses to indicate the relative viability of alternative karyotypes.

In females, we used data from large bottle populations of F_1_ flies from our experiment on XXY frequency change, described below (equivalent to S2 Fig). Crosses designed to detect secondary non-disjunction in XXY females (S4 and S5 Figs) also indicate viability effects of the XYY male karyotype, relative to XY males; in these assays, XYY and XY can be distinguished due to the fact that the Y chromosome of the female parent is marked but that of the male parent is not. We analyzed offspring frequencies as binomial responses using a GLMM accounting for a random effect of block and using an individual-level random effect to account for overdispersion, calculating Wald confidence intervals. These tests will underestimate XYY viability effects if there is non-disjunction in males or secondary non-disjunction in XXY females (see S4 Fig), but as both rates are low, we expect this bias to be negligible.

### Female reproductive traits

We examined several reproductive traits in XXY females (collected as described above), in comparison with XX females. We made some comparisons with both XX females collected from the same vials as the XXY females (which will therefore differ in marker phenotype, see S2 Fig), or with XX females collected from separate vials, resulting from a cross of *y*/*y* females with wild-type males, which will have the same marker phenotype as the focal XXY females (+/*y*, non-*yellow*).

We assessed the attractiveness of individual focal females (XXY or XX) in mate choice trials with a competitor female and a wild-type male. The competitor females carried the *brown* eye color marker, *bw*/*bw*, allowing us to identify whether the focal or competitor female was the first to mate. We first placed the females in one vial and the male in another vial under CO_2_ anesthesia, and then combined the male and females without anesthesia to begin the mate choice assay. We generally discarded any trials in which no mating occurred within approximately 200 min. Females from these trials were also included in subsequent fecundity assays, with egg counts proceeding immediately for females who copulated in the attractiveness assay. We analyzed the frequency of trials in which the focal female was the first to mate using a chi-squared test and tested for among-group variation in mating latency using a Kruskal-Wallis rank sum test.

To assess fecundity, we placed focal females (2–5 days post-eclosion) individually in vials with two wild-type males for one day, and then discarded the males and transferred the females without anesthesia to new vials in time increments of 16 h or 8 h; these vials contained standard media plus blue food coloring, facilitating egg counts at each transfer. We counted all eggs produced over four days and retained the oviposition vials; we counted adults from these vials after 11 and 15 days to estimate offspring production. We also scored the survival of focal females during the assay period; females used for the fecundity assay were also included in measurements of dry body mass (see below). We analyzed these traits using Kruskal-Wallis rank sum tests. Additionally, we compared body mass between XX and XXY females bearing different marker phenotypes but reared in the same vials, using a t-test.

### Dry mass

To measure dry body mass, we first froze flies individually in small tubes at –20C for at least one day. Next, we opened the tubes and dried flies at 70C for approximately 24h. Finally, we weighed dried flies individually on a Mettler Toledo XP105DR analytical balance (d = 0.01 mg). In some cases, mass measurements were collected for females that were also involved in fecundity measurements; in these cases, the female was frozen at the end of the fecundity assay (146 cases), or shortly after death (11 cases).

### Egg size

To examine egg size, we placed petri dishes of standard media seeded with live yeast in cages of 130–160 flies and collected eggs after overnight oviposition. We measured eggs laid by XXY females or XX females reared in the same vials (differentiated by body color phenotype) when placed in cages with Dp(1;Y)*y*^+^ males. We also measured eggs from wild-type flies as a standard for comparison. Finally, we also measured eggs from marked and non-marked females reared in the same vials (S8 Fig) to test for an effect of the *y* marker. We estimated egg length (*L*) and width (*W*) under 100 × magnification using a calibrated stage micrometer and calculated egg volume as (1/6)π*W*^2^*L*, which assumes a spheroid shape [78].

### Development time

We examined egg to adult development time using the cross in S2 Fig, which will produce equal frequencies of XX and XXY flies (distinguishable by body color) in the absence of viability selection. We allowed two XXY females and two Dp(1;Y)*y*^+^ males to produce offspring in each of 26 vials for three days, and then scored the emergence of adult offspring twice daily for the next 18 days. We also scored the emergence times of males, among which XY and XYY karyotypes are expected but not phenotypically distinguishable in this case. For analysis we treated each fly as a replicate, with an emergence time equal to the midpoint of the observation period in which they emerged.

### Maternal effects

Differences between XXY females derived from spontaneous non-disjunction versus those derived from XXY parents could arise if there are maternal effects of the XXY karyotype. To examine this possibility, we designed crosses to obtain XX females with the *y*/*y* marker genotype from either XXY parents or XX parents (S9 Fig). We used a similar design to obtain XY males with the *y*/Y marker genotype from either XXY parents or XX parents (S9 Fig). We measured the reproductive success of these focal flies in replicate vials consisting of two focal flies, two *bw*/*bw* flies of the same sex as the focal flies, and four *bw*/*bw* flies of the opposite sex as the focal flies. Additionally, we performed body mass measurements on focal flies using the approach described above.

### XYY male reproductive success

We also obtained XYY males to assess their reproductive success (S10 Fig), compared with XY males collected separately (in both cases the males have wild-type body color). We generated replicate vials of two focal males (either XYY or XY), two competitor *bw*/*bw* males and four *bw*/*bw* females each. We analyzed the reproductive success of focal males, indicated by the proportion of non-brown-eyed offspring, using a quasibinomial generalized linear model.

### XXY frequency change

To learn about the overall fitness impact of the XXY and XYY karyotypes, we allowed karyotype frequencies to evolve in experimental populations. We initiated three populations using 60 XXY females and 60 XY males each and maintained them in bottles for 12 generations. In these populations, it was possible to distinguish female karyotypes based on body color. The female karyotype frequencies at generation 1 (i.e., the first generation of offspring) were used to infer the viability effect of XXY (S2 Fig). In subsequent generations, the frequency of XXY females will reflect their own fitness as well as that of XYY males and additional karyotypes. Finding no evidence for variation among populations, we analyzed these data as binomial responses using a GLM. We also conducted matching Wright-Fisher-like simulations of XXY frequency change over 12 generations, and tested whether simulated populations with and without selection against XYY males resembled our observed data, considering the slope associated with generation in a binomial GLM.

### Standing frequency of XXY

We used two approaches to estimate the standing frequency of the XXY karyotype in an outbred laboratory population of the Canton-S background, in which the Y chromosome is un-marked. In the first approach, we sampled females as virgins from the lab population and performed a test cross to males with the compound X-Y chromosome C(1;Y)2, *y*^1^ *B*^1^. If the female is XX, all un-marked male offspring of this cross will be X-null and sterile, whereas half of the un-marked male offspring of XXY females will be XY and fertile (S11 Fig; in the absence of secondary non-disjunction). For each female sampled, we tested the fertility of several male offspring, typically in two vials with three males each, housed with several *bw*/*bw* females; the *bw* marker ensures that females that are non-virgin due to experimental error would produce offspring that are distinguishable from those of fertile focal males. We performed these tests on 775 male offspring produced by 158 females of unknown karyotype.

Additionally, we sampled females from the laboratory population and performed PCR to test for Y chromosome presence. We extracted DNA from female heads to avoid any influence of Y-bearing sperm that might be present in the reproductive tract, and digested using protease K. We used the following primer sequences (5’ to 3’) to amplify part of the Y-linked gene kl-2, with an expected product size of 409 bp: AGTTATTTCCCAACCGCGTG, CAGGAAGTCGCGCACATTTA. Our preliminary tests confirmed that these primers produce detectable bands when applied to XY males or known XXY females from the marked-Y strain. To reduce the risk of false positives due to sample contamination we also included negative controls in the form of XX females from the marked-Y strain and excluded any blocks of data in which bands were detected in these samples. The retained data consists of 75 Canton-S females of unknown karyotype, 40 positive controls (30 known XXY females plus 10 XY males) and 30 negative controls (XX females from the marked-Y stock).

After establishing that the XXY frequency estimates from the two methods above were statistically indistinguishable (see Results), we analyzed the combined results using ABC to obtain a single frequency estimate and account for the potential for false negatives under the test cross method (i.e., the probability that a sample of males would not include any fertile males if they were present at 50% frequency in a given family). We drew random values for the standing frequency *f* from the uniform distribution *U*(0, 0.1). For a given simulation reflecting the test cross assay, *n*_1_ = *B*(158, *f*) females were assigned the XXY karyotype, where *B* is the binomial distribution. The number of detected cases is then $n_{1}^{\prime }=\sum_{n_{1}}sgn\;B(k_{i},\;0.5)$, where *sgn* is the sign function and *k*_*i*_ is the number of male offspring tested for female *i*, sampled from the dataset (ranging from 1 to 6, mean 4.9). For a given simulation reflecting the PCR assay, *n*_2_ = *B*(75, *f*) females were assigned the XXY karyotype. We retained 50000 simulated values of *f* that produced $n_{1}^{\prime }$ = 1 and *n*_2_ = 1 as the posterior distribution and report the mean and 95% credible interval of this distribution based on quantiles.

### Model of mutation-selection balance for aneuploidy

We modeled the deterministic frequency dynamics of XX and XXY females and XY and XYY males under random mating, incorporating our estimates of sex-specific nondisjunction rates, the viability impact of the XYY karyotype, and the standing frequency of the XXY karyotype. The unknown parameters are the frequency of XYY males and the relative fitness of the XXY karyotype. We numerically solved for the steady-state karyotype frequencies, producing estimates of the unknown parameters. We sampled 50000 values from the posterior distributions of the known parameters to obtain credible intervals for the unknown parameters. Details and equations for karyotype frequency change are given in S3 Text.

## Supporting information

S1 Text Estimating sex-specific non-disjunction rates by maximum likelihood. (DOCX)

S2 Text Expected effects of spontaneous alterations to chromosome marking. (DOCX)

S3 Text Model of mutation-selection balance for aneuploidy. (DOCX)

S1 Fig Production of XXY females via spontaneous nondisjunction. The products of the specified cross are indicated, including in cases of nondisjunction. Some cells are shaded to highlight visible phenotypes and inviability.
(TIF)

S2 Fig Production of XXY females and XYY males from existing XXY flies. The products of the specified cross are indicated, including in cases of nondisjunction. Some cells are shaded to highlight visible phenotypes and inviability. Males with additional Y chromosomes can potentially be identified based on the Hairy-wing (Hw) phenotype.
(TIF)

S3 Fig Detection of nondisjunction and chromosome loss in females and males. The products of the specified cross are indicated, including in cases of nondisjunction. Some cells are shaded to highlight visible phenotypes and inviability. Chromosome symbols are as in S2 Fig.
(TIF)

S4 Fig Products of XXY females crossed to WT males and estimation of XYY male viability. The products of the specified cross are indicated, including in cases of nondisjunction. Some cells are shaded to highlight visible phenotypes and inviability.
(TIF)

S5 Fig Products of XXY females crossed to marked males and estimation of XYY male viability. The products of the specified cross are indicated, including in cases of nondisjunction. Some cells are shaded to highlight visible phenotypes and inviability.
(TIF)

S6 Fig Products of XXY females crossed to C(1;Y) males and estimation of X∅ and XXYY viability. The products of the specified cross are indicated, including in cases of nondisjunction. Some cells are shaded to highlight visible phenotypes and inviability.
(TIF)

S7 Fig Identification of XYY males and offspring produced. The products of the specified cross are indicated, including in cases of nondisjunction. Some cells are shaded to highlight visible phenotypes and inviability.
(TIF)

S8 Fig Testing the effect of a phenotypic marker on viability. The products of the specified cross are indicated, including in cases of nondisjunction. Some cells are shaded to highlight visible phenotypes.
(TIF)

S9 Fig Estimating maternal effects of the XXY karyotype on reproductive success. We performed a set of crosses to obtain male and female flies with shared karyotypes and markers but alternative maternal karyotypes; we measured the reproductive success of these flies in competition with *bw*/*bw* flies.
(TIF)

S10 Fig Obtaining XYY males for testing. The products of the specified cross are indicated, including in cases of nondisjunction. Some cells are shaded to highlight visible phenotypes and inviability. In this cross, XYY males can be identified even in the presence of secondary nondisjunction in females; non-*y* XY males can also arise nondisjunction in males, but this will be very rare.
(TIF)

S11 Fig Test cross for identifying XXY females in an un-marked strain. In a cross with C(1;Y) males, XX females will produce male offspring that are all sterile, whereas XXY females will produce some fertile male offspring.
(TIF)

S1 Table Production of XXY females via spontaneous non-disjunction, where parent of origin cannot be distinguished. See S1 Fig.
(XLSX)

S2 Table Production of exceptional progeny via spontaneous non-disjunction and chromosome loss, where parent of origin can be determined. See S3 Fig.
(XLSX)

S3 Table Products of XXY females in a cross with WT males. See S4 Fig.
(XLSX)

S4 Table Products of XXY females in a cross with m^D^ males. See S5 Fig.
(XLSX)

S5 Table Products of XXY females in a cross with C(1;Y) males. See S6 Fig.
(XLSX)

S6 Table Identification of XYY sires and resulting offspring. See S7 Fig.
(XLSX)

S7 Table Test for viability effect of yellow marker on males and females. See S8 Fig.
(XLSX)

S8 Table Frequency change of the XXY karyotype in three populations over 12 generations. Generation 1 data used to infer XXY viability effect.
(XLSX)

S9 Table Characteristics of XX vs. XXY females: mating, mass, reproduction and survival. Some flies, but not all, were included in multiple trait assays.
(XLSX)

S10 Table Measures of dry body mass for XX and XXY females reared together, distinguishable by marker phenotype. See S2 Fig.
(XLSX)

S11 Table Size of eggs producted by females of different karyotype and marker phenotypes. (XLSX)

S12 Table Development time in XXY females (non-yellow), XX females (yellow) and males. Start time of vials 11/28/22 19:30.
(XLSX)

S13 Table Maternal effects of XX versus XXY on the reproductive success of male and female offspring. See S9 Fig.
(XLSX)

S14 Table Maternal effects of XX versus XXY on the dry mass of male and female offspring. See S9 Fig.
(XLSX)

S15 Table Reproductive success of XYY males in competition with bw/bw males. (XLSX)

S16 Table Assay for standing frequency of XXY based on test crosses. See S11 Fig.
(XLSX)

S1 File R code used for data analysis. (R)
