# Supplementary material for: Fitness consequences of sex chromosome aneuploidy in Drosophila melanogaster
Source: PLoS Genet. 2025 Jun 3;21(6):e1011703. doi: 10.1371/journal.pgen.1011703 (PMC12133181; doi:10.1371/journal.pgen.1011703)
Supplement: S1 Text — (DOCX) [file pgen.1011703.s001.docx]

**S1 Text**

*Estimating sex-specific non-disjunction rates by maximum likelihood*

We considered both the number of exceptional flies observed in a test cross where female and male non-disjunction can be distinguished (including cases of non-disjunction in both parents; S3 Fig and S2 Table), and the number of exceptional (XXY) females observed in a cross where the parent of origin cannot be determined (S1 and S1 Table). Exceptional females (XXY) can appear only due to non-disjunction, whereas exceptional males (X-null) can appear due to either non-disjunction or spontaneous chromosome loss. We maximized the following function using the *mle2* function [78]  with the Nelder-Mead optimization method:

*log*(*B*(*N*_f🡪f_ | *n* = *N*_f_, *p* = ½ *µ*_female_)) +

*log*(*B*(*N*_m🡪f_ | *n* = *N*_f_, *p* = *µ*_male_)) +

*log*(*B*(*N*_f🡪m_ | *n* = *N*_m_, *p* = ½ *µ*_female_ + ½ 𝜆_female_)) +

*log*(*B*(*N*_m🡪m_ | *n* = *N*_m_, *p* = *µ*_male_ + 𝜆_male_)) +

*log*(*B*(*N*_fm🡪f_ | *n* = *N*_f_, *p* = ½ *µ*_female_ *µ*_male_ + ½ *µ*_female_ 𝜆_male_)) +

*log*(*B*(*N*_fm🡪m_ | *n* = *N*_m_, *p* = ½ *µ*_female_ *µ*_male_ + ½ *µ*_male_ 𝜆_female_)) +

*log*(*B*(*N*^*^_XXY_ | *n* = *N*^*^_f_, *p* = ½ *µ*_female_ + *µ*_male_))

where *B* is the binomial probability mass function; *N_b_*_🡪_*_a_* is the number of exceptional flies of sex *a* originating from a parent of sex *b* (f = female, m = male, fm = both parents); *N_c_* is the total number of offspring of sex *c*; *µ_c_* is the rate of non-disjunction in sex *c*; 𝜆*_c_* is the rate of chromosome loss during meiosis in sex *c*. In the final term, *N*^*^_XXY_ and *N*^*^_f_ refer to the number of XXY females and total females, respectively, observed in the cross where the non-disjunction parent of origin cannot be determined. Note that when non-disjunction occurs in females, half of the exceptional progeny of a given sex will be inviable, so ½ *µ*_female_ is used. This model does not consider second-order phenomena such as chromosome loss in aneuploids and should be considered approximate.

To test the hypothesis that rates of female and male non-disjunction differ, we repeated the maximum-likelihood optimization with *µ*_female_ and *µ*_male_ replaced with *µ*_common_, and compared models using a likelihood ratio test. We used the same approach to test for a difference in chromosome loss rates between female and male-derived chromosomes.
