# Supplementary material for: Fitness consequences of sex chromosome aneuploidy in Drosophila melanogaster
Source: PLoS Genet. 2025 Jun 3;21(6):e1011703. doi: 10.1371/journal.pgen.1011703 (PMC12133181; doi:10.1371/journal.pgen.1011703)
Supplement: S2 Text — (DOCX) [file pgen.1011703.s002.docx]

**S2 Text**

*Expected effects of spontaneous alterations to chromosome marking*

We considered whether the low rate of secondary nondisjunction we observed could be an artifact due to the presence of a *y*^+^ allele on an X chromosome, perhaps due to a rare X-Y recombination event or reversion of the *y*^1^ marker. Several observations are inconsistent with this possibility. First, if stocks of putative XXY females (*y*^1^/*y*^1^/*y*^+^) were actually XX (*y*^1^/*y*^+^ or *y*^+^/*y*^+^), we would expect the non-yellow phenotype to increase in frequency, as females can become homozygous for the wild-type *y*^+^ allele; instead, we saw that both the yellow and non-yellow phenotypes remained present in roughly equal frequency over multiple generations. Second, in the presence of an X-linked *y*^+^ allele, a test cross of males to *y*^1^/*y*^1^ females would produce only non-yellow female offspring; instead, we confirmed that yellow females result from such a cross. Third, our cross of putative XXY females to wild-type males (Fig. S4) produced fewer non-yellow males than yellow males, indicating viability selection against the XYY karyotype; the presence of an X-linked *y*^+^ allele in this cross would cause the opposite pattern, i.e., more non-yellow males than yellow males. Finally, we used putative XXY females as positive controls for our PCR-based assay of standing XXY frequency, and in so doing confirmed the presence of Y-linked DNA in these females.
