# Supplementary material for: Fitness consequences of sex chromosome aneuploidy in Drosophila melanogaster
Source: PLoS Genet. 2025 Jun 3;21(6):e1011703. doi: 10.1371/journal.pgen.1011703 (PMC12133181; doi:10.1371/journal.pgen.1011703)
Supplement: S3 Text — (DOCX) [file pgen.1011703.s003.docx]

**S3 Text**

*Model of mutation-selection balance for aneuploidy*

We modeled the deterministic frequency dynamics of XX and XXY females and XY and XYY males, assuming that other sex chromosome configurations are inviable and unobserved, and that the sex ratio remains even. We considered the frequency of the XXY karyotype among all females (*f*_XXY_ = 1 – *f*_XX_) and their fitness relative to XX females (*w*_XXY_, where *w*_XX_ = 1), and the frequency of XYY males (*f*_XYY_ = 1 – *f*_XY_) and their fitness relative to XY males (*w*_XYY_, where *w*_XY_ = 1). We defined sex chromosome nondisjunction rates for males and females as *µ*_male_ and *µ*_female_ respectively and considered the possibility of higher rates of nondisjunction in aneuploid flies (“secondary” nondisjunction; *µʹ*_male_ and *µʹ*_female_). For given current frequencies of XXY females and XYY males, we determined the expected frequencies of XXY females and XYY males in the following generation (*t* + 1). Following standard formulation, these are found as

*f*_XXY_(*t* + 1) = *p*_XXY_ *w*_XXY_ / *w*_F_ [eqn. 1]

and

*f*_XYY_(*t* + 1) = *p*_XYY_ *w*_XYY_ / *w*_M_ [eqn. 2]

Here, *w*_F_ = *w*_XX_ *p*_XX_ + *w*_XXY_ *p*_XXY_, and *w*_M_ = *w*_XY_ *p*_XY_ + *w*_XYY_ *p*_XYY_.

The values of *p* are the expected frequencies of progeny, given the current frequencies *f*(*t*) and the nondisjunction rates *µ*, before the application of selection. To find values for *p*, we assume random mating and consider probabilities of nondisjunction among all possible matings. The resulting equations are shown below in an un-simplified form to better illustrate the outcomes of different mating events.

*p*_XX_ = *f*_XX_ *f*_XY_ [½ (1 – *µ*_female_) (1 – *µ*_male_) + ½ *µ*_female_ ½ *µ*_male_]

+ *f*_XXY_ *f*_XY_ [½ (1 – *µʹ*_female_) ½ (1 – *µ*_male_) + ½ *µʹ*_female_ ½ *µ*_male_]

+ *f*_XX_ *f*_XYY_ [(1 – *µʹ*_female_) ½ *µ*_male_]

+ *f*_XXY_ *f*_XYY_ [½ (1 – *µʹ*_female_) ½ *µʹ*_male_]

*p*_XXY_ = *f*_XX_ *f*_XY_ [(1 – *µ*_female_) ½ *µ*_male_ + ½ *µ*_female_ ½ (1 – *µ*_male_)]

+ *f*_XXY_ *f*_XY_ [½ (1 – *µʹ*_female_) ½ (1 – *µ*_male_) + ½ (1 – *µʹ*_female_) ½ *µ*_male_ + ½ *µʹ*_female_ ½ (1 – *µ*_male_)]

+ *f*_XX_ *f*_XYY_ [(1 – *µ*_female_) ½ (1 – *µʹ*_male_) + ½ *µ*_female_ ½ (1 – *µʹ*_male_)]

+ *f*_XXY_ *f*_XYY_ [(1 – *µʹ*_female_) ½ (1 – *µʹ*_male_) + ½ (1 – *µʹ*_female_) ½ *µʹ*_male_ + ½ *µʹ*_female_ ½ (1 – *µʹ*_male_)]

*p*_XY_ = *f*_XX_ *f*_XY_ [(1 – *µ*_female_) ½ (1 – *µ*_male_) + ½ *µ*_female_ ½ *µ*_male_]

+ *f*_XXY_ *f*_XY_ [½ (1 – *µʹ*_female_) ½ (1 – *µ*_male_) + ½ (1 – *µʹ*_female_) ½ *µ*_male_ + ½ *µʹ*_female_ ½ (1 – *µ*_male_)]

+ *f*_XX_ *f*_XYY_ [(1 – *µ*_female_) ½ (1 – *µʹ*_male_) + ½ *µ*_female_ ½ (1 – *µʹ*_male_)]

+ *f*_XXY_ *f*_XYY_ [½ (1 – *µʹ*_female_) ½ (1 – *µʹ*_male_) + ½ *µʹ*_female_ ½ *µʹ*_male_]

*p*_XYY_ = *f*_XX_ *f*_XY_ (0)

+ *f*_XXY_ *f*_XY_ [½ (1 – *µʹ*_female_) ½ (1 – *µ*_male_) + ½ *µʹ*_female_ ½ *µ*_male_]

+ *f*_XX_ *f*_XYY_ [(1 – *µ*_female_) ½ *µʹ*_male_]

+ *f*_XXY_ *f*_XYY_ [½ (1 – *µʹ*_female_) ½ *µʹ*_male_ + ½ *µʹ*_female_ ½ (1 – *µʹ*_male_)]

We set *f*_XY_ = 1 – *f*_XYY_ and *f*_XX_ = 1 – *f*_XXY_, *w*_XX_ = 1 and *w*_XY_ = 1. We assume we have estimated values for *µ*_male_, *µ*_female_, *µʹ*_male_ and *µʹ*_female_, as well as *f*_XXY_ and *w*_XYY_. This leaves *f*_XYY_ and *w*_XXY_ as the only unknowns, so we can solve for the steady-state values numerically using [eqn. 1] and [eqn. 2] by setting *f*(*t* + 1) = *f*(*t*) for each karyotype. We initially assumed *µʹ*_male_ = *µ*_male_, and *µʹ*_female_ = *µ*_female_, since our data suggest that rates of secondary nondisjunction may be similar to rates of initial nondisjunction. Secondarily, we explored the effect of higher rates of secondary nondisjunction by using representative values from the literature: *µʹ*_male_ = *µʹ*_female_ = 0.032 [14].
