# Supplementary figures and images for: Fitness consequences of sex chromosome aneuploidy in Drosophila melanogaster
Source: PLoS Genet. 2025 Jun 3;21(6):e1011703. doi: 10.1371/journal.pgen.1011703 (PMC12133181; doi:10.1371/journal.pgen.1011703)

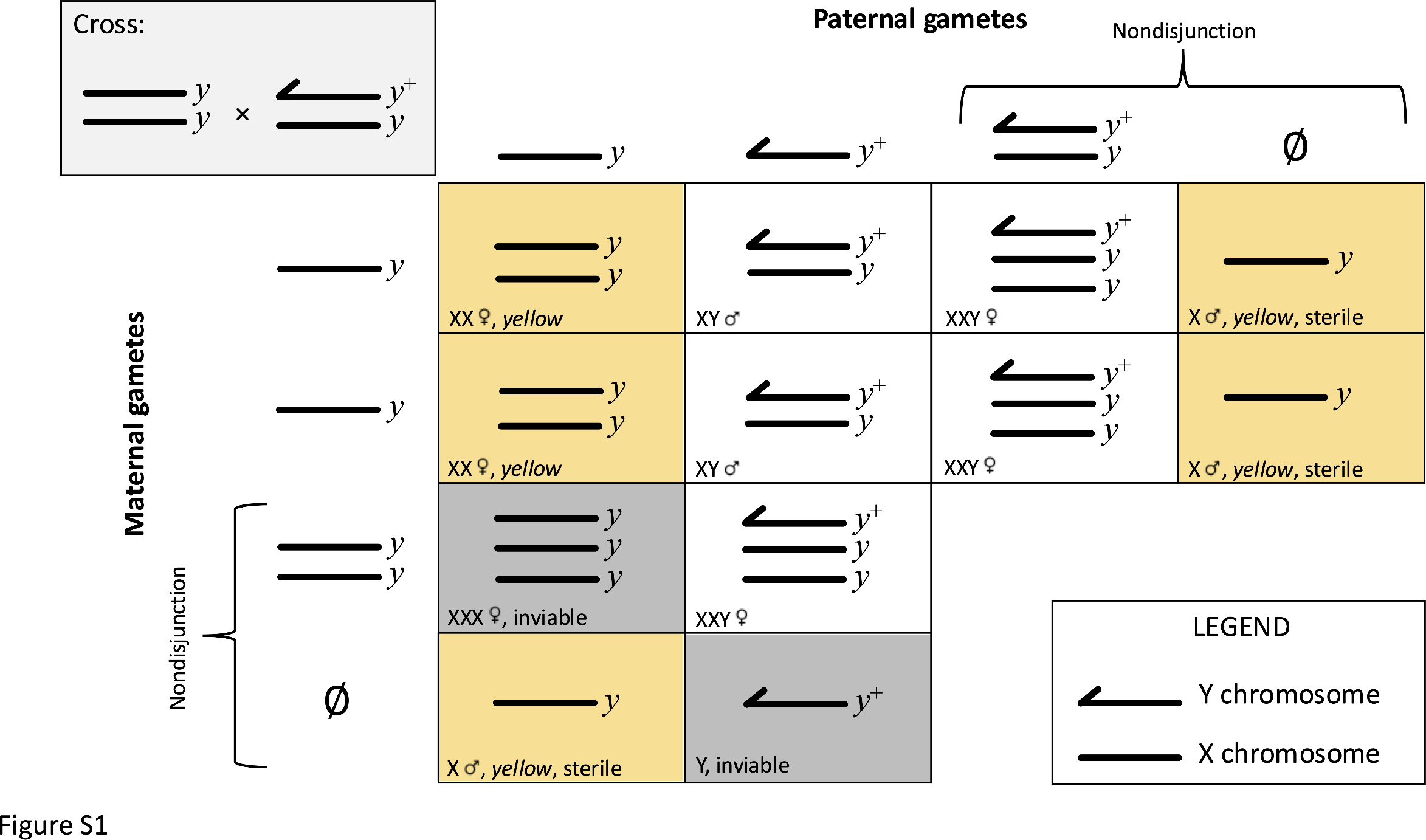

Supplement: S1 Fig — The products of the specified cross are indicated, including in cases of nondisjunction. Some cells are shaded to highlight visible phenotypes and inviability. (TIF) [file pgen.1011703.s004.tif]

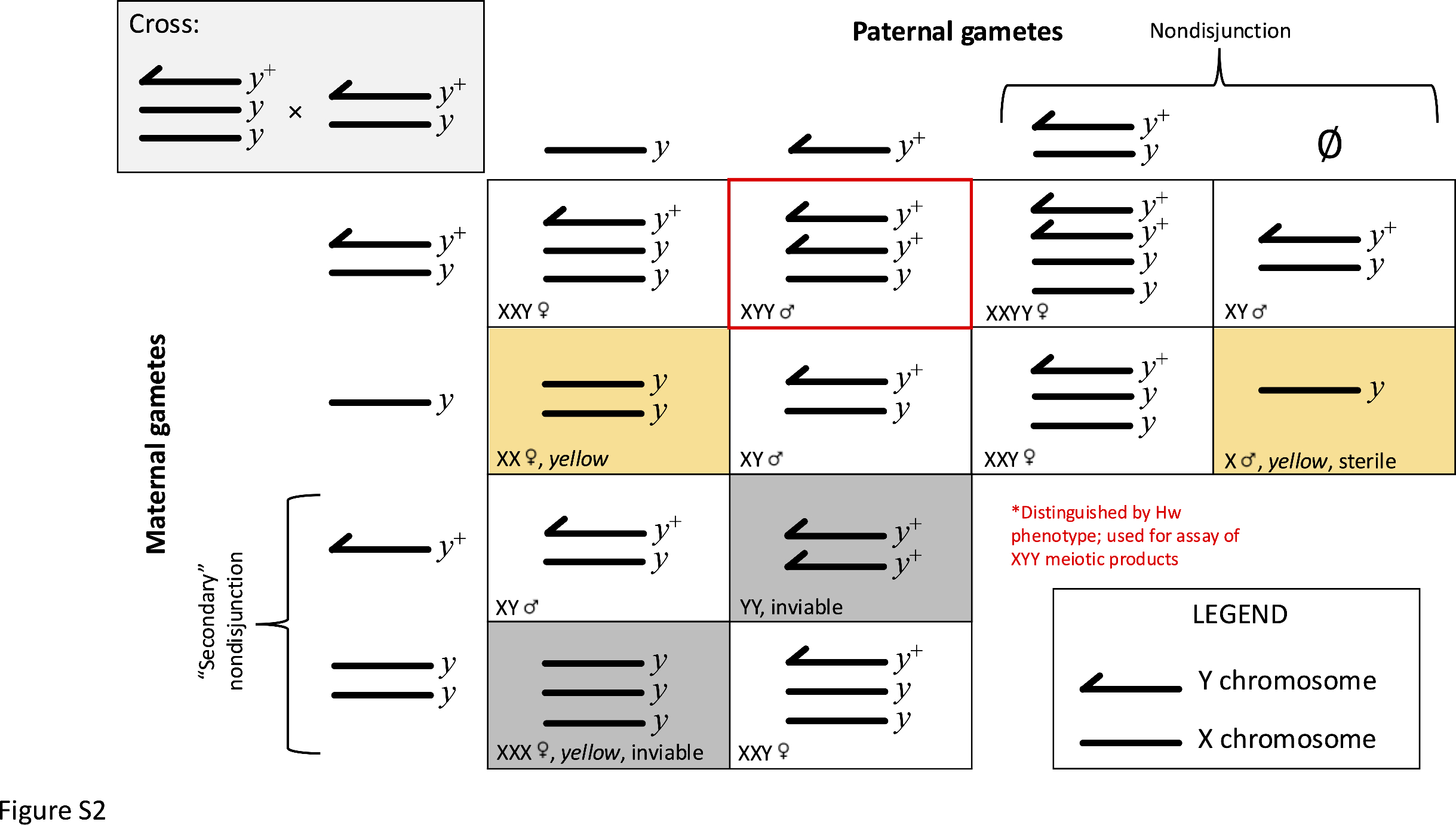

Supplement: S2 Fig — The products of the specified cross are indicated, including in cases of nondisjunction. Some cells are shaded to highlight visible phenotypes and inviability. Males with additional Y chromosomes can potentially be identified based on the Hairy-wing (Hw) phenotype. (TIF) [file pgen.1011703.s005.tif]

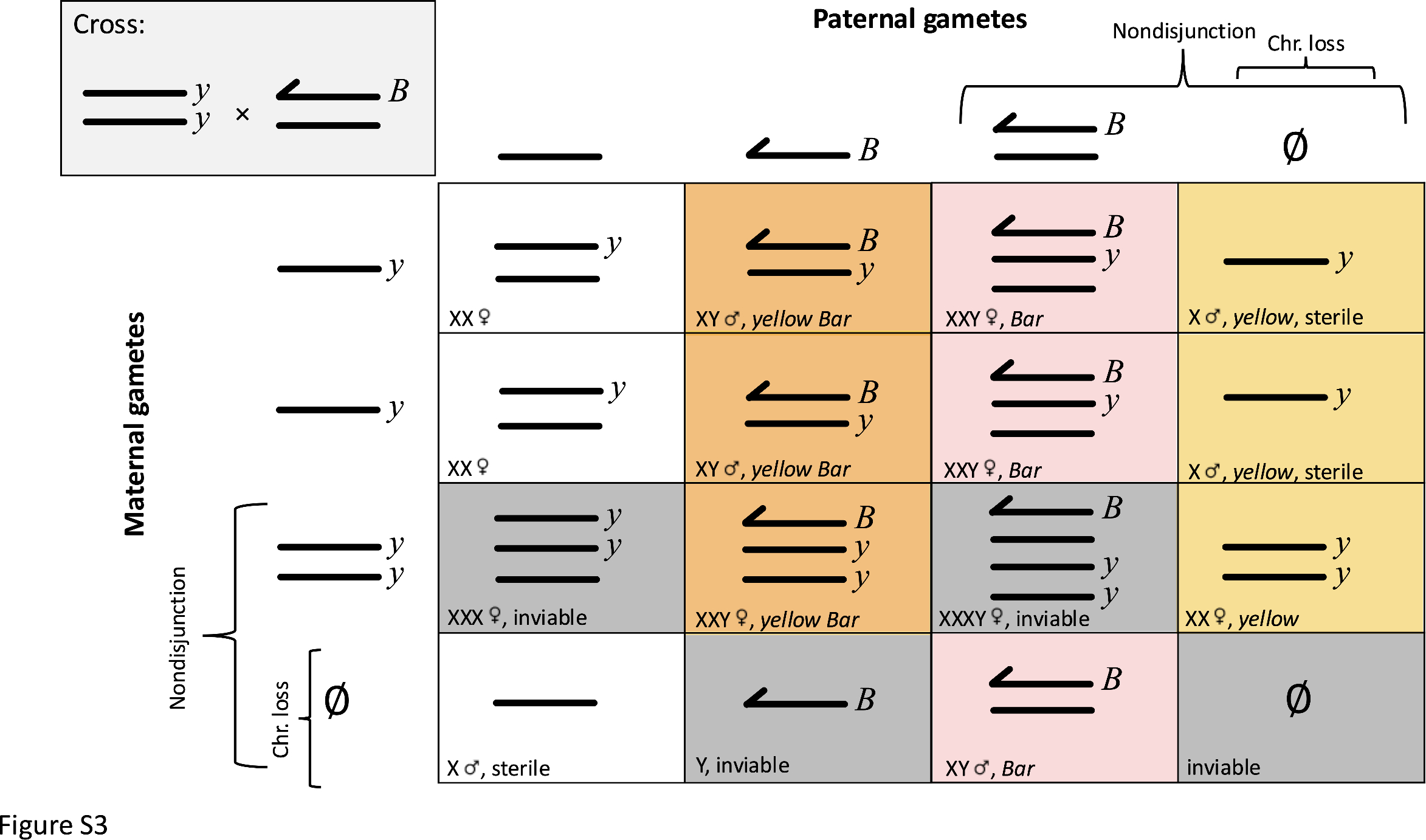

Supplement: S3 Fig — The products of the specified cross are indicated, including in cases of nondisjunction. Some cells are shaded to highlight visible phenotypes and inviability. Chromosome symbols are as in S2 Fig. (TIF) [file pgen.1011703.s006.tif]

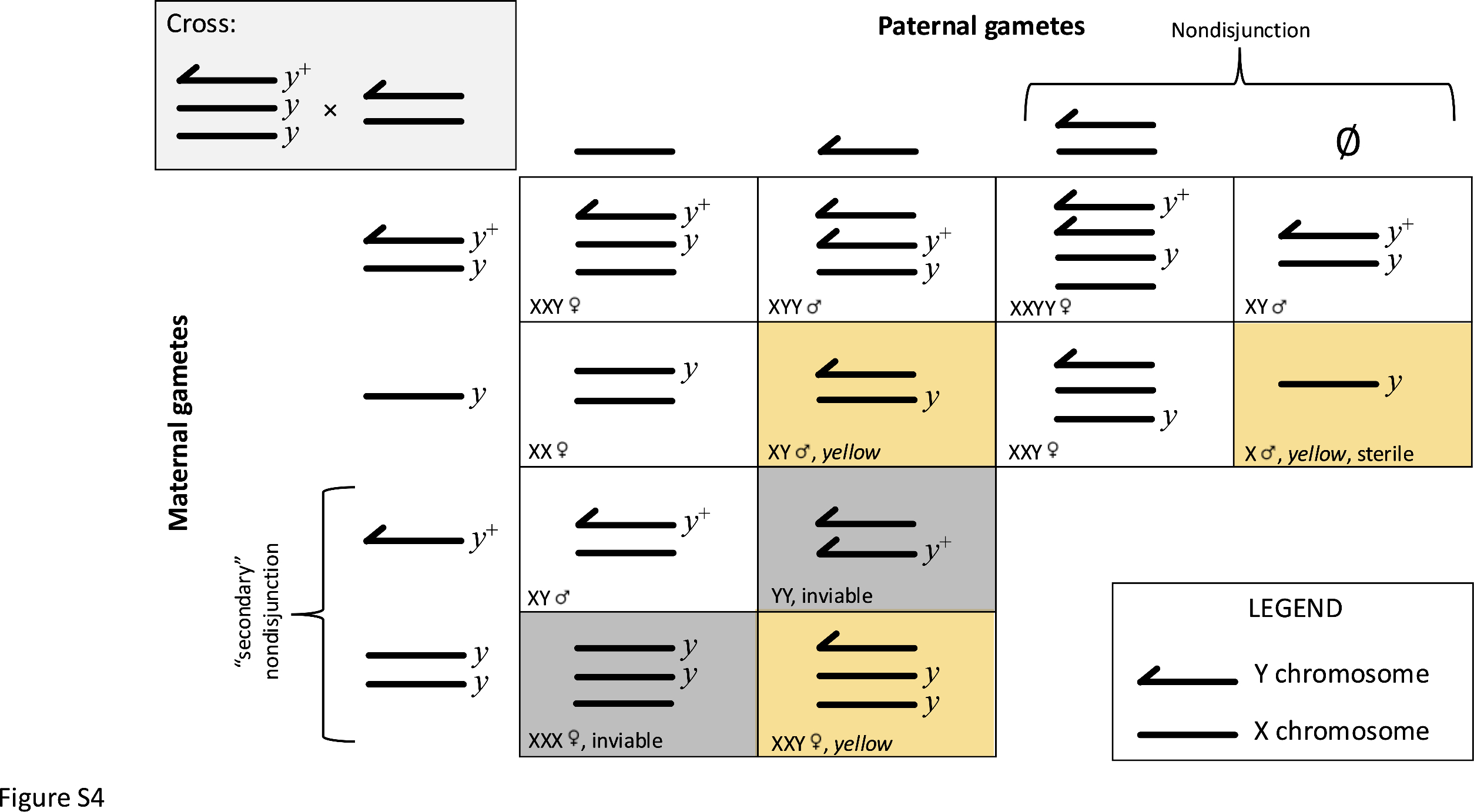

Supplement: S4 Fig — The products of the specified cross are indicated, including in cases of nondisjunction. Some cells are shaded to highlight visible phenotypes and inviability. (TIF) [file pgen.1011703.s007.tif]

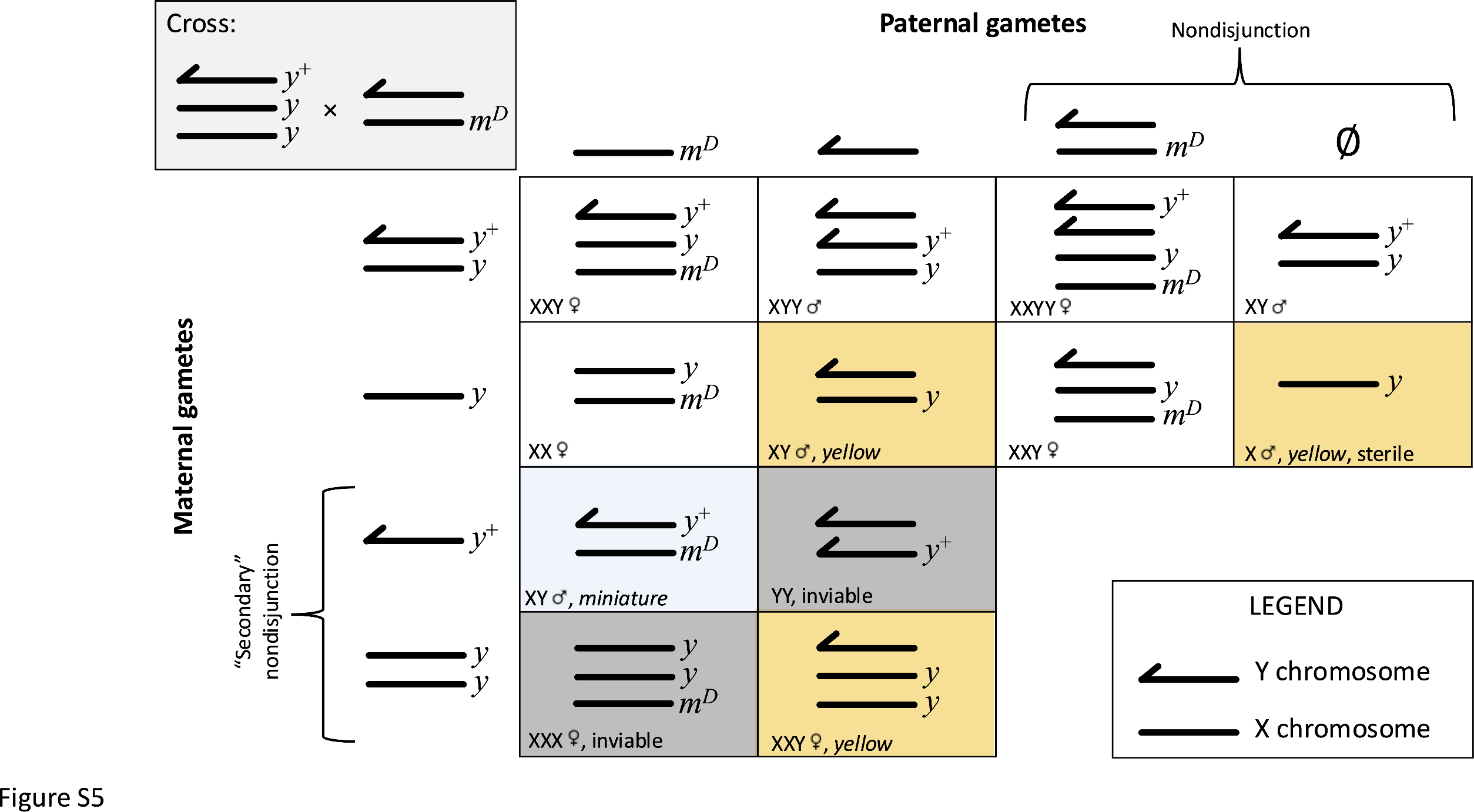

Supplement: S5 Fig — The products of the specified cross are indicated, including in cases of nondisjunction. Some cells are shaded to highlight visible phenotypes and inviability. (TIF) [file pgen.1011703.s008.tif]

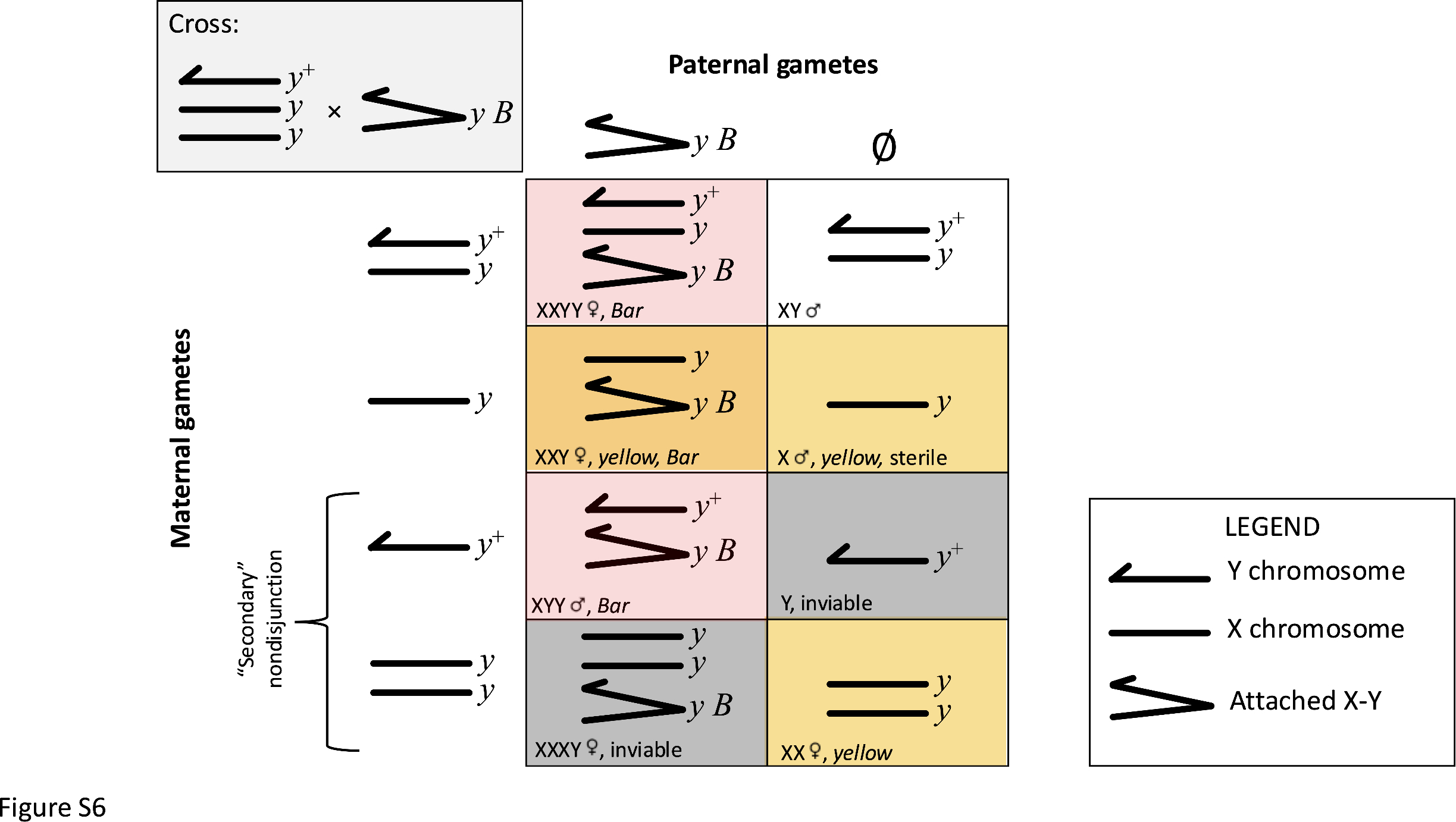

Supplement: S6 Fig — The products of the specified cross are indicated, including in cases of nondisjunction. Some cells are shaded to highlight visible phenotypes and inviability. (TIF) [file pgen.1011703.s009.tif]

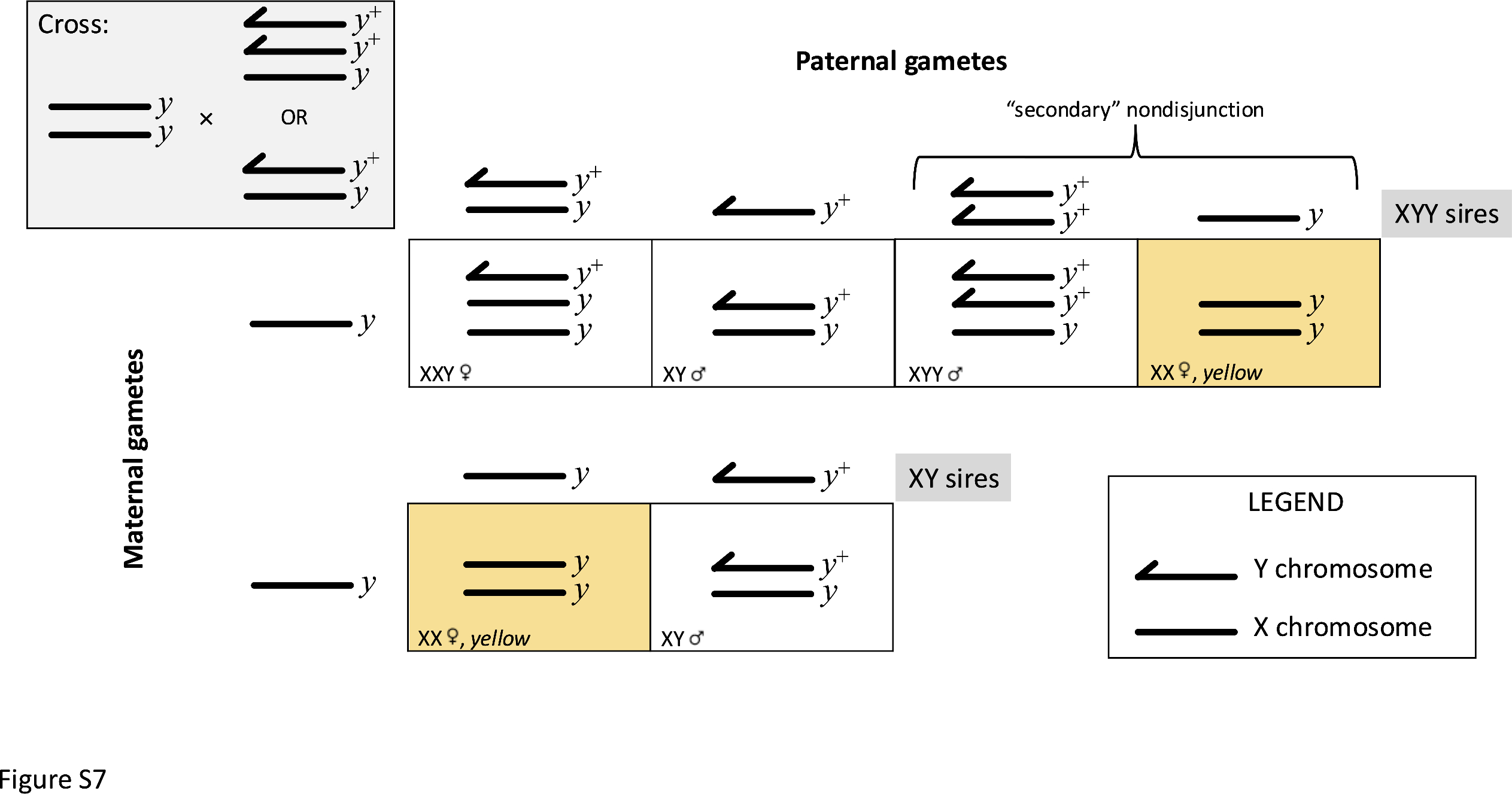

Supplement: S7 Fig — The products of the specified cross are indicated, including in cases of nondisjunction. Some cells are shaded to highlight visible phenotypes and inviability. (TIF) [file pgen.1011703.s010.tif]

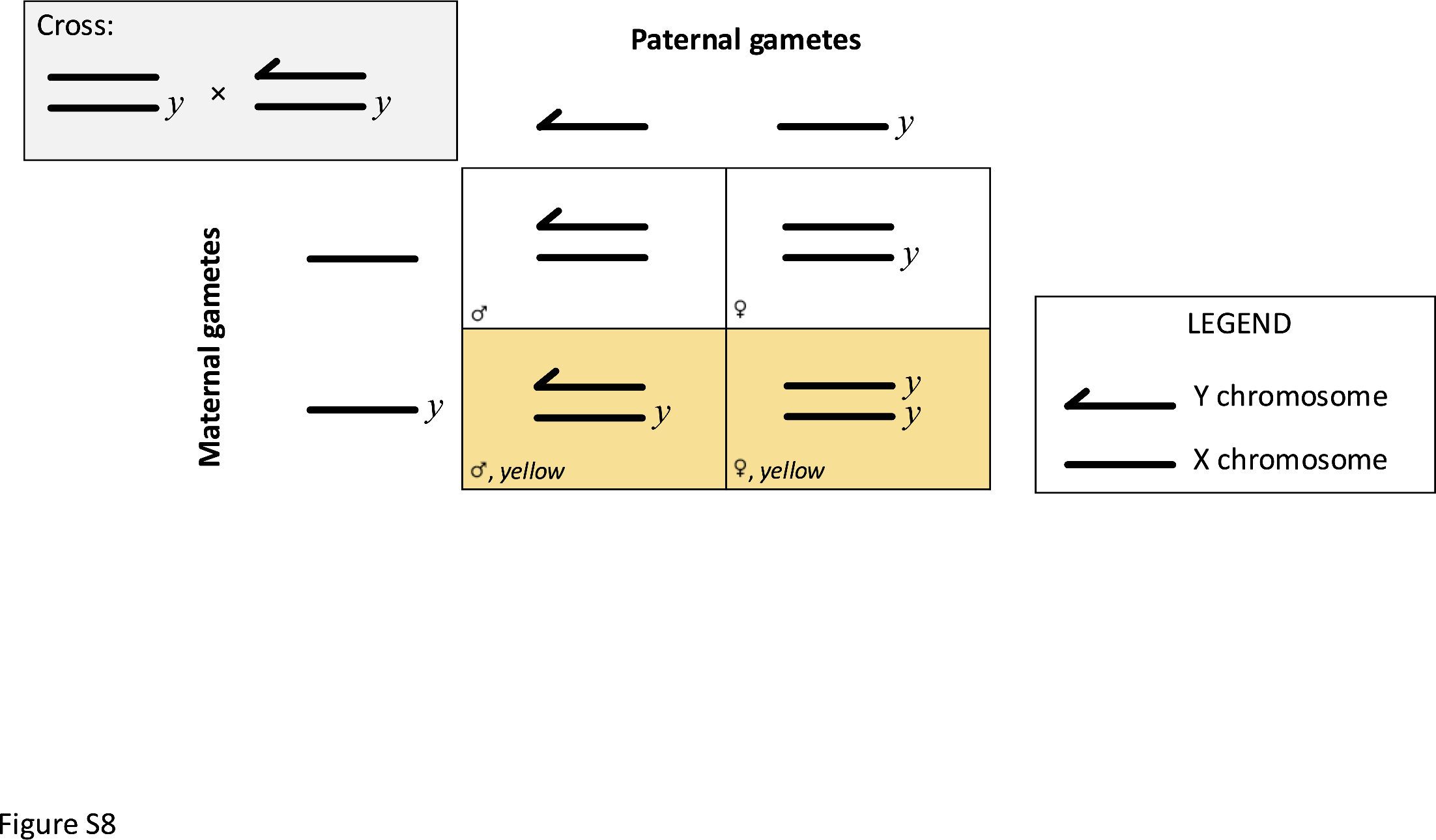

Supplement: S8 Fig — The products of the specified cross are indicated, including in cases of nondisjunction. Some cells are shaded to highlight visible phenotypes. (TIF) [file pgen.1011703.s011.tif]

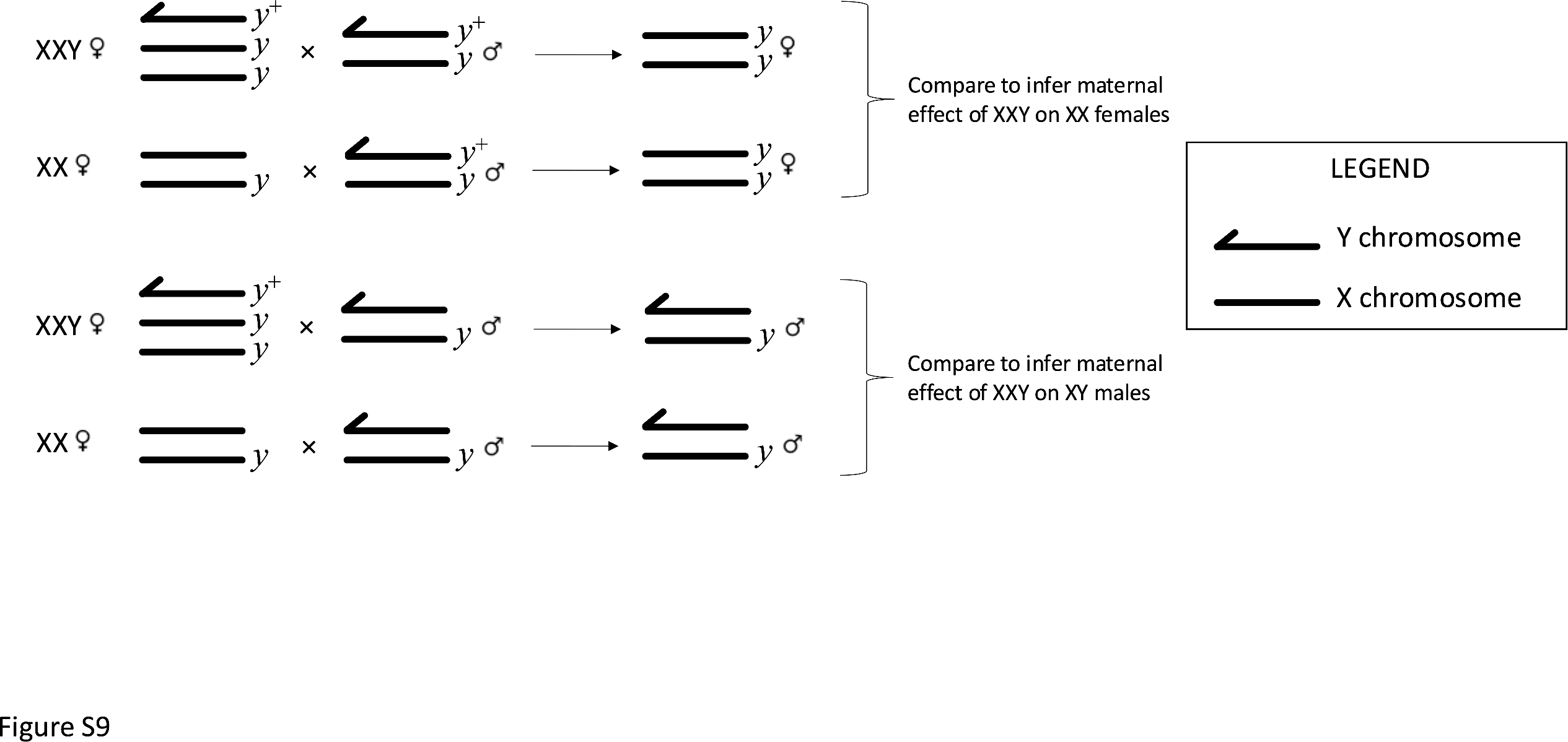

Supplement: S9 Fig — We performed a set of crosses to obtain male and female flies with shared karyotypes and markers but alternative maternal karyotypes; we measured the reproductive success of these flies in competition with bw/bw flies. (TIF) [file pgen.1011703.s012.tif]

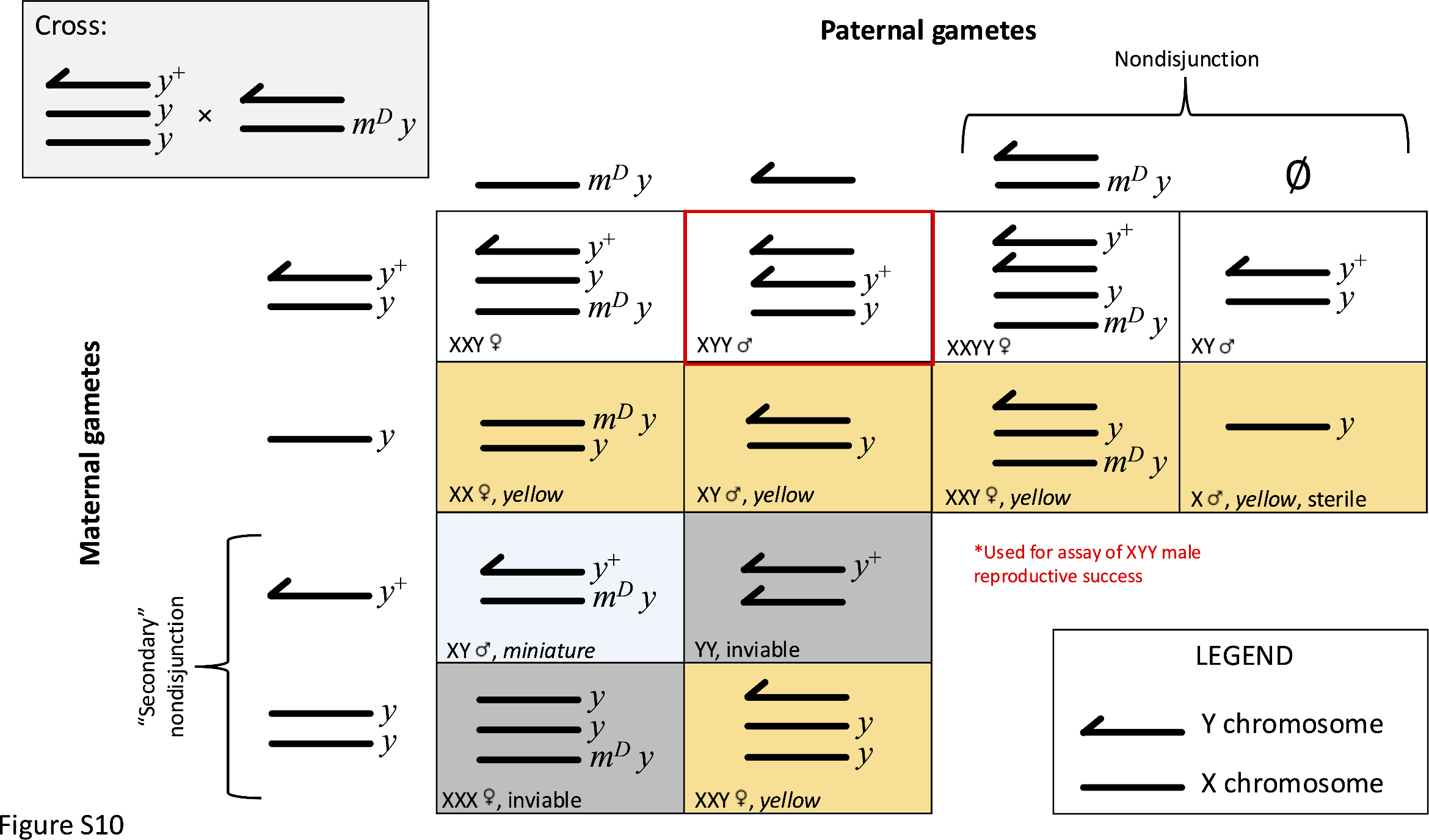

Supplement: S10 Fig — The products of the specified cross are indicated, including in cases of nondisjunction. Some cells are shaded to highlight visible phenotypes and inviability. In this cross, XYY males can be identified even in the presence of secondary nondisjunction in females; non-y XY males can also arise nondisjunction in males, but this will be very rare. (TIF) [file pgen.1011703.s013.tif]

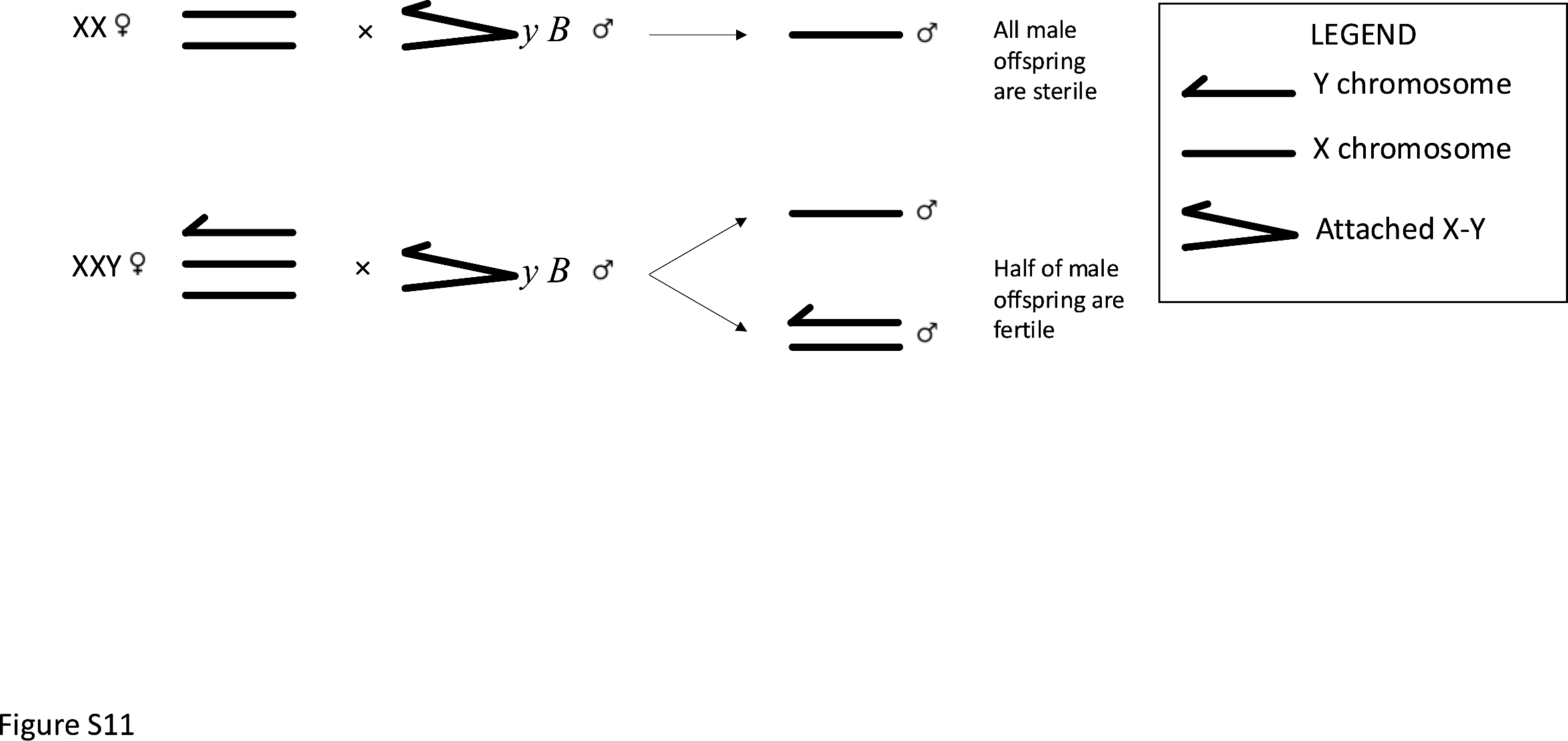

Supplement: S11 Fig — In a cross with C(1;Y) males, XX females will produce male offspring that are all sterile, whereas XXY females will produce some fertile male offspring. (TIF) [file pgen.1011703.s014.tif]
